# Identification of Alternatively-Activated Pathways between Primary Breast Cancer and Liver Metastatic Cancer Using Microarray Data

**DOI:** 10.3390/genes10100753

**Published:** 2019-09-25

**Authors:** Limei Wang, Jin Li, Enze Liu, Garrett Kinnebrew, Xiaoli Zhang, Daniel Stover, Yang Huo, Zhi Zeng, Wanli Jiang, Lijun Cheng, Weixing Feng, Lang Li

**Affiliations:** 1 College of Automation, Harbin Engineering University, Harbin 150001, China; 2 Department of Biomedical Informatics, College of Medicine, The Ohio State University, Columbus, OH 43210, USA; 3 The Center for Computational Biology and Bioinformatics, School of Medicine, Indiana University, Indianapolis, IN 46202, USA

**Keywords:** alternatively-activated pathway, breast cancer, liver metastasis, microarray, gene active status

## Abstract

Alternatively-activated pathways have been observed in biological experiments in cancer studies, but the concept had not been fully explored in computational cancer system biology. Therefore, an alternatively-activated pathway identification method was proposed and applied to primary breast cancer and breast cancer liver metastasis research using microarray data. Interestingly, the results show that *cytokine-cytokine receptor interaction* and *calcium signaling* were significantly enriched under both conditions. *TGF beta signaling* was found to be the hub in network topology analysis. In total, three types of alternatively-activated pathways were recognized. In the *cytokine-cytokine receptor interaction* pathway, four active alteration patterns in gene pairs were noticed. Thirteen cytokine-cytokine receptor pairs with inverse activity changes of both genes were verified by the literature. The second type was that some sub-pathways were active under only one condition. For the third type, nodes were significantly active in both conditions, but with different active genes. In the *calcium signaling* and *TGF beta signaling pathways*, node E2F5 and E2F4 were significantly active in primary breast cancer and metastasis, respectively. Overall, our study demonstrated the first time using microarray data to identify alternatively-activated pathways in breast cancer liver metastasis. The results showed that the proposed method was valid and effective, which could be helpful for future research for understanding the mechanism of breast cancer metastasis.

## 1. Introduction

Cancer is a leading cause of death in the world. Breast cancer is the leading cause of cancer-related death among females worldwide. In 2012, an estimated 1.7 million cases and 521,900 deaths occurred [1]. Cancer lethality is mainly caused by metastasis, accounting for over 90% of human cancer deaths [2]. Despite its clinical importance, little is known about the genetic and biochemical determinants of metastasis. 

Understanding the nature of the genes involved in metastasis has been an important goal for the past several decades. Along with the development of microarray and other high-throughput sequencing technologies, plenty of new prognostic markers (metastasis signatures or differentially-expressed genes) that predict metastasis risk in patients with breast cancer were reported [3]. A set of 70 genes (MammaPrint DX) was identified to predict the likelihood of distant metastases in young patients in a retrospective study [4]. The 70-gene signature was validated in several independent studies, such as Van de Vijver [5], Bueno-de-Mesquita et al. [6], Buyse et al. [7], Mook et al. [8,9], Wittner et al. [10], and Curtis [11]. Another 76-gene signature was retrospectively reported to be able to predict the outcome in patients of all age groups with lymph-node-negative breast cancer using a different microarray platform [12]. In these studies, the gene signatures were proven to be more powerful than traditional clinical parameters (the St Gallen criteria [13] and the National Institutes of Health criteria [14]). In addition, many other studies also reported metastasis gene signatures, including Ma et al. [15] (2 genes), Pail et al. [16] (21 genes), Landemaine et al. [17] (6 genes), and Patsialou et al. [18] (Human Invasion Signiture, HIS, 445 transcripts, of which 185 were annotated protein-genes). While these prognostic markers, i.e., signatures, are extremely valuable in predicting breast cancer metastasis, they were not studied for identifying drug targets or developing new treatment strategies for metastatic breast cancer.

In this paper, we used microarray data collected from public domain resources and studied the similarities and differences of molecular pathways between breast cancer primary tumor and breast cancer liver metastasis samples. Biological changes that occur during the metastatic progression of breast cancer are still incompletely characterized [19]. Pathway analysis strategies have been widely used in cancer research and identified some metastasis-related pathways, such as in breast cancer [20,21,22], pancreatic ductal adenocarcinoma [23], and colorectal cancer [24,25]. In this study, we are not only interested in the molecular pathways that are enriched in the breast cancer liver metastasis tumors, but also the alternatively-activated pathways when the breast cancer primary tumors metastasize to the liver. The alternatively-activated pathway is defined as the activated pathway in different states, such as primary tumors vs. metastasis tumors, tumors with or without treatment. In those conditions, there are different differentially-expressed/-enriched genes, or different pathway/sub-pathway topologies, or different active sub-pathways. Many alternatively-activated/rewired pathways in cancers via biological experiments were detected, such as the cell polarity signaling pathway [26], apoptotic signaling networks in anticancer drugs enhances cell death research [27], and alternatively-activated ERK-JNK signaling pathways in melanoma [28]. The time course was used mechanistically to detect alternatively-activated pathways (dynamical system models) under each condition in the Q-method [29]. However, this alternatively-activated pathway concept has not been fully explored using the computational cancer system biology method.

Common approaches to identify pathways or gene expression signatures or any other list of differentially-expressed genes include individual gene analysis (IGA) and gene set analysis (GSA) [30,31]. Both methods are used to investigate whether the identified gene signatures are statistically enriched with genes from predefined pathways. In IGA techniques, such as the Database for Annotation, Visualization and Integrated Discovery (DAVID) [32,33], there are mainly two steps. The first step is to identify significantly differentially-expressed genes (DEGs) either between different groups or conditions such as between a primary cancer group and a metastatic group. The over-representation of these DEGs in specific pathways is then determined by counting statistics, such as Fisher’s exact test or Chi squared analysis. In contrast, GSA techniques, such as Gene Set Enrichment Analysis (GSEA) [34], take all genes in a predefined pathway and calculate a gene-level statistic for each gene. Next, a pathway-level statistic is derived by combining the gene-level statistics, and to determine whether the pathway represented by the gene set is significantly perturbed. Other pathway analyses integrated the topology of signaling pathways into their enrichment analyses [35,36]. However, none of these existing pathway analyses can be directly applied to identify our hypothesized alternatively-activated pathways that happen when the breast cancer primary tumors metastasize to the liver. 

Although a number of breast cancer metastasis pathways were identified [37,38], their regulatory mechanisms and differences between primary tumor and metastatic tumor have not been described. Understanding the altered regulatory mechanisms can aid us in understanding and eventually treating metastatic breast cancer. 

In this paper, we developed a novel pathway enrichment analysis method that can identify differentially-enriched pathways between two conditions using microarray data. Notably, it can identify alternatively-activated pathways when primary tumors metastasize to distal sites. To demonstrate its application, breast cancer liver metastasis tumors were used to investigate the uniquely enriched pathways and alternatively-activated pathways compare to primary tumors.

## 2. Materials and Methods

The microarray data for primary breast cancer and metastasis were downloaded from the Gene Expression Omnibus (GEO) database, and genes were first classified as active or inactive based on present/margin/absent (PMA) information. Next, the alternatively-activated gene pathways were calculated based on the proposed method. Finally, the pathway networks were used to better understand the identified alternatively-activated pathways, and the 3 top selected different types of alternatively-activated pathways were further analyzed.

### 2.1. Gene Expression and Pathways

The gene expression microarray data were download from the GEO database. One hundred fifty three samples were in the primary breast cancer group [39] (GSE65194, Affymetrix Human Genome U133 Plus 2.0 Array), and 43 samples were in the breast cancer metastasized to liver group [40,41] (GSE46141: 16 samples, and GSE56493: 27 samples, Rosetta/Merck Human RSTA Custom Affymetrix 2.0 microarray). GSE65194 contains 130 breast cancer samples (41 TNBC; 30 Her2; 30 Luminal B and 29 Luminal A) and 23 technical duplicates. As there was a sample imbalance between the primary cancer group and metastatic cancer group, we performed a bootstrap analysis. Each time, 43 samples were randomly selected from the primary cancer group and compared to the 43 samples from the metastatic cancer group. Pathway analysis was then performed based on the DEGs. We repeated this 10 times. 

A total of 186 KEGG pathways and 676 REACTOME pathways were downloaded from MsigDB v6.0 [34] and used for the pathway analysis.

### 2.2. Gene Expression Data Pretreatment

The R package “affy” was used to analyze the retrieved CEL files containing the raw microarray data. We calculated the PMA information (present/margin/absent) for each probe and transformed it to the gene expression status. A probe with P (present) status means that this probe is active in this sample and will be kept for further analysis. If any probe for a gene was active, the gene was considered as active. Genes without an active probe were considered inactive.

### 2.3. Breast Cancer Liver Metastasis Uniquely-Enriched Pathway Analysis

**Figure 1:**
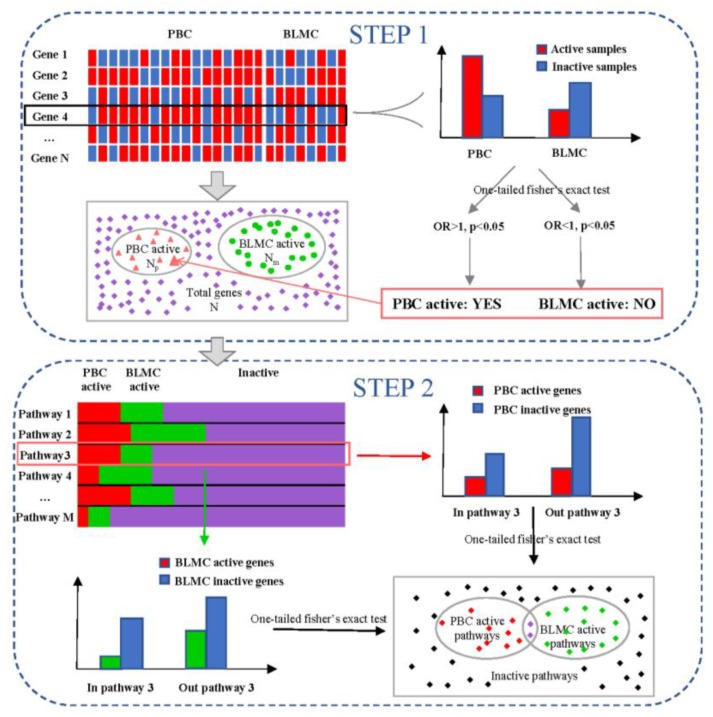
Flowchart.

To determine alternatively-activated pathways in liver metastasis, we used an IGA-like two-step method. In Step 1, we identified the differentially-expressed active genes; and in Step 2, we identified the differentially-activated pathways (Figure 1).

Step 1: For each gene, we calculated the number of active samples in the primary cancer group (denoted as $n_{11}$), the number of inactive samples in the primary cancer group (denoted as $n_{12}$), the number of active samples in the metastatic cancer group (denoted as $n_{21}$), and the number of inactive samples in the metastatic cancer group (denoted as $n_{22}$). Both the left and right one-tailed Fisher’s exact tests were performed. To test whether a gene was more active in primary cancer than in metastatic cancer (left one-tailed test), the hypothesis is:

(1) $$H_{0}:\;p_{1}=p_{2}\;H_{1}:\;p_{1}>p_{2}$$

To test whether a gene was more active in metastatic cancer than in primary cancer (right one-tailed test), the hypothesis is:

(2) $$H_{0}:\;p_{1}=p_{2}\;H_{1}:\;p_{1}<p_{2}$$

where $\;p_{1}$ is the proportion of active samples in primary cancer ($n_{11}$/$(n_{11}$+$n_{12})$) and $p_{2}$ is the proportion of active samples in metastatic cancer ($n_{21}$/$(n_{21}$+$n_{22})$). 

After these 2 tests with a threshold *p* < 0.05, the genes were divided into 3 sets: primary-cancer-active, metastatic-cancer-active, and no significant differential activity between primary cancer and metastatic cancer. In the proposed method, these significant primary-cancer-active and metastatic-cancer-active genes were considered as differentially-activated genes.

Step 2: for each pathway, two one-tailed Fisher’s exact tests were performed to test whether this pathway was more active in primary cancer than in metastatic cancer or more active in metastatic cancer than primary cancer, or not significantly different between primary cancer and metastatic cancer. Suppose the numbers of primary-cancer-active genes, metastatic-cancer-active genes, and total genes in a pathway are $m_{p}$, $m_{m}$, m  and the numbers of primary-cancer-active genes, metastatic-cancer-active, and total genes are $N_{p}$, $N_{m}$, N. The hypothesis in the first one-tailed Fisher’s exact test is:

(3) $$H_{0}:\;p_{3}=p_{4}\;H_{1}:\;p_{3}>p_{4}$$

where $p_{3}$ is the proportion of primary-cancer-active genes in this pathway ($\frac{m_{p}}{m}$) and $p_{4}$ is the proportion of primary-cancer-active genes out of this pathway ($\frac{N_{p}-m_{p}}{N-m}$).

The hypothesis in the second one-tailed Fisher’s exact test is:

(4) $$H_{0}:\;p_{5}=p_{6}\;H_{1}:\;p_{5}>p_{6}$$

where $p_{5}$ is the proportion of metastatic-cancer-active genes in this pathway ($\frac{m_{m}}{m}$) and $p_{6}$ is the proportion of metastasis-cancer-active genes out of this pathway ($\frac{N_{m}-m_{m}}{N-m}$).

We used *p* < 0.05 as the significance threshold for both tests.

### 2.4. Gene-Gene Activity Alteration Patterns’ Recognition

**Figure 2:**
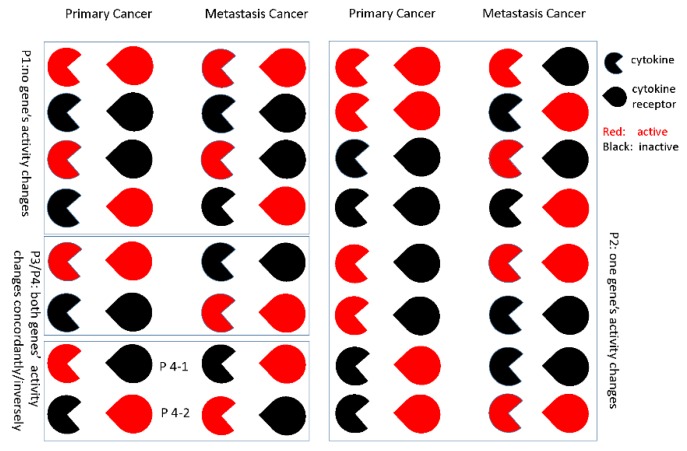
Gene-gene activity alteration patterns.

Even in a significant metastasis-related pathway, not all the gene-gene regulations are changed. For each gene-gene pair, there are different activity alteration patterns between primary cancer and metastatic cancer. We defined 4 types of activity alterations as follows. The cytokine-cytokine receptor relationships are shown as an example in Figure 2.

Pattern 1: no gene’s activity changes

Pattern 2: one gene’s activity changes

Pattern 3: both genes’ activity changes concordantly 

Pattern 4: both genes’ activity changes inversely 

For each gene-gene pair, we used Fisher’s exact test to determine activity alteration patterns. If the genes related to metastasis were not in Pattern 1, we tested Patterns 2–4. For a specific gene-gene pair, taking Pattern 4 as an example, suppose $S_{1}$ samples with the active-inactive state and $S_{2}$ samples with the inactive-active state are in the primary cancer group and $S_{3}$ samples with the active-inactive state and $S_{4}$ samples with the inactive-active state are in the metastatic cancer group; a left-tailed Fisher’s exact test was performed to test Pattern 4-1, and a right-tailed Fisher’s exact test was performed to test Pattern 4-2. In these tests, we used a *p* < 0.05 as the significance threshold. For each gene-gene pair, there may be several significant active alteration patterns. We selected the pattern with the minimal *p*-value as the regulated alteration pattern because the pattern with the minimal *p*-value may be the most possible pattern. Here, we are more interested in Pattern 4.

### 2.5. Bayesian Network Construction

Several networks and network-based approaches have been widely used in cancer research, such as human interactome data [42] and gene co-expressed protein interaction networks [43]. In the human interactome, although some gene interactions can be directed, most of them are used as an undirected network in the current network-based methods. A Bayesian network is a probabilistic network that takes a group of random variables and their conditional dependencies to represent them as a directed acyclic graph (DAG). To identify a network structure that best interprets the input, we used the “greedy equivalence search” (GES) method, a score-based algorithm that generates a pattern that represents the optimal causal network [44]. With the assumption that the true regulatory network is a DAG, the greedy search algorithm sequentially applies two phases: a “forward phase” and a “backward phase”, where the “forward phase” provides a pattern that includes the optimal network, while the “backward phase” returns a pattern that is equivalent to the optimal network. The algorithm will terminate when no more edges can be added to or removed from the graph. In this study, we used the fast greedy search (FGS) algorithm [45], which is an optimized and parallelized implementation of GES. FGS was used to build a directed gene network (DGN) for primary breast cancer and breast to liver metastasis data. FGS algorithm is mainly controlled by two parameters that can affect the density of the network: penalty discount and depth. The penalty discount is the BIC score that FGS uses to evaluate networks. Depth refers to the degree constraint of each node. We chose a low penalty discount score and unlimited degree constraint (PD = 6, depth = −1) to allow high degree nodes.

### 2.6. Expand Pathways with the Bayesian Network

Bayesian networks may provide novel relationships among genes, not available in the pathway databases. They are directed networks that are different from the protein interaction networks or gene co-expressed networks. We could get the upstream or downstream gene information from the Bayesian network, even though these gene relationships may be potentially regulated. We expanded each pathway using the downstream genes in the Bayesian networks. The expanded primary/metastatic cancer pathways should have a higher probability to be active in the primary/metastatic cancer situation and more possible with specific biological meanings. 

**Figure 3:**
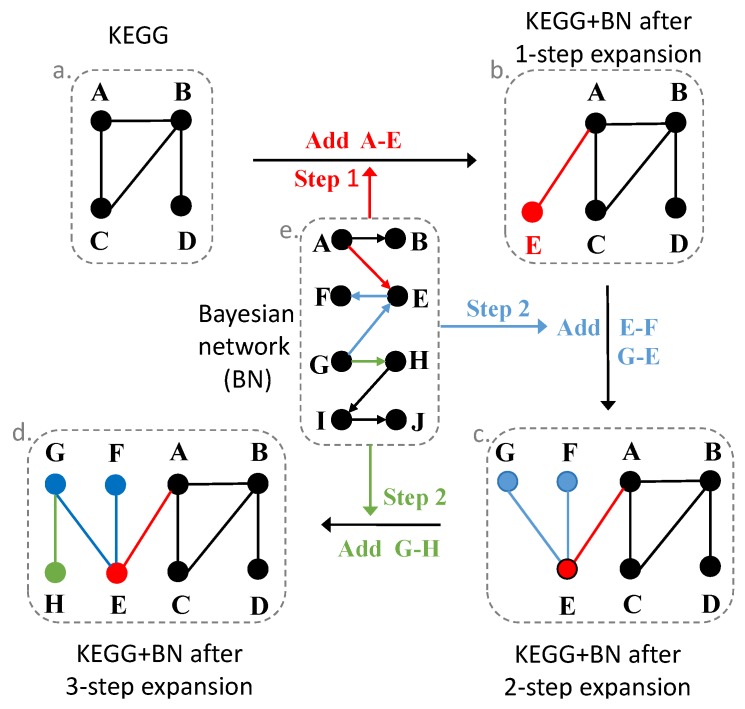
KEGG pathways expansion with the Bayesian network.

We expanded each pathway with two Bayesian networks constructed from primary breast cancer data and breast to liver metastatic cancer data separately (Figure 3). For gene I in a pathway, if gene J regulates I or is regulated by I in the Bayesian network, gene J will be added into this pathway. After we expanded all the genes in each pathway using Bayesian networks, we got two new pathway gene sets. We repeated this procedure 3 times, and finally, we obtained 7 pathway gene sets (1 raw pathway gene set, 3 expanded pathway gene sets with the primary cancer Bayesian network, and 3 expanded pathway gene sets with the metastatic cancer Bayesian network).

### 2.7. Connectivity among Pathways: Pathway Networks

To further explore the connectivities among pathways, we constructed a pathway network based on Fisher’s exact tests (hypergeometric distribution test) between pathways. Suppose there are $n_{all}$ genes in all the pathways, $n_{a}$ genes in Pathway A, $n_{b}$ genes in Pathway B, and $n_{ab}$ genes in both Pathways A and B; the *p*-value of this test is calculated by:

(5) $$p=1-\sum_{i=1}^{n_{ab}-1}\frac{C_{n_{a}}^{i}C_{n_{all}-n_{a}}^{n_{b}-i}}{C_{n_{all}}^{n_{b}}}$$

After calculating the *p*-value, we performed multiple tests’ adjustment using the FDR method in R (“fdr” in the “p.adjust” function) [46]. An FDR <0.05 was chosen as the threshold. After the test, we retained all the significant pairs between differentially-active pathways and constructed the pathway network using Cytoscape [47]. We only constructed the pathway network for KEGG pathways. First, we constructed pathway networks using genes in each pathway from the database, and we called it the general condition. Next, we constructed pathway networks based on genes in extended pathways, and we called it the primary breast cancer or breast cancer liver metastasis condition. Lastly, we compared these networks and determined primary breast cancer-specific pathway connections and breast to liver metastatic specific pathway connections.

## 3. Results

### 3.1. Raw Pathway Analysis

Out of the 186 KEGG pathways, 17 pathways were significantly enriched among primary breast cancer active genes, and 24 pathways were significantly enriched among breast cancer liver metastasis active genes. Interestingly, two pathways, *cytokine-cytokine receptor interaction* (hsa04060) and *calcium signaling* (hsa04020), were significantly enriched under both conditions. The *p*-values and statistics of these 186 pathways are in Supplemental Table S1.

Out of the 676 REACTOME pathways, 39 pathways were significantly enriched among primary breast cancer active genes, and 81 pathways were significantly enriched among breast cancer liver metastasis active genes. Two pathways, *chemokine receptors bind chemokines* (R-HSA-380108) and *peptide ligand-binding receptors* (R-HSA-375276), were significantly enriched under both conditions. *Chemokine receptors bind chemokines* is a subset of *peptide ligand-binding receptors*. 

Chemokine receptors are cytokine receptors found on the surface of certain cells, which interact with a type of cytokine called chemokines. This REACTOME pathway, *chemokine receptors bind chemokines* R-HAS-380108, is highly related to the KEGG pathway *cytokine-cytokine receptor interaction* has04060. Therefore, we further analyzed the cytokine-cytokine receptor interaction pathway in the next step. 

### 3.2. Robustness Analysis

As there were about 3.5-times more samples in the primary cancer group than the metastatic cancer group, we performed robustness analysis for the group imbalance. We randomly selected 43 samples from the primary cancer group and performed pathway analysis with the 43 metastatic cancer samples. This procedure was repeated 10 times. 

First, we compared the active genes between the primary and metastatic samples. Of these, 1481/4523 primary/metastatic cancer active genes were identified based on all 153/43 samples, and on average, 1163.4/3895.6 primary/metastatic cancer active genes were found using the random balanced 43/43 samples. We obtained 78%/86% active genes using random samples compared to using all the samples, and most of the active genes (number: 1131.1/3839.9, proportion: 97.41%/98.58%) in the random analysis can be found from the analysis using all samples. The detailed results are in Supplemental Table S2.

After obtaining the active genes, we compared the KEGG pathways. The 17/23 primary/metastatic cancer active pathways were found using all 153/43 samples, and on average, 11.9/23.3 primary/metastatic cancer active pathways were identified using the random 43/43 samples. Most of the active pathways (number: 10.6/21.3, proportion: 89.38%/91.44%) in the random analysis were the same from the analysis using all the samples. The results using different data were highly correlated, with an average Pearson correlation coefficient of 0.9035 and 0.9633 for primary cancer active pathways and metastatic cancer pathways, respectively. The pathways *cytokine-cytokine receptor interaction* and *calcium signaling* can be found in most of the results from random samples. The detailed results are in Supplemental Table S3.

Through the active gene and pathway robustness analysis for group imbalance, the proposed method was robust for the sample size imbalance. 

### 3.3. Expanded Network

After expanding the pathways with Bayesian networks, the numbers of genes in each pathway increased exponentially (Supplemental Figure S1). The number of significantly differentially-active pathways was also greatly increased (Table 1). Basically, the primary cancer Bayesian network contained more information about genes active in primary cancer, and the metastatic cancer Bayesian network contained more information about genes active in metastatic cancer. Therefore, we obtained more significant primary-active pathways in primary-expanded networks and more metastasis-active pathways in metastasis-expanded networks. From Table 1, we can see that the more steps we expanded, the more significant results we obtained. After two or more steps, the influence of the Bayesian network was much greater than the raw pathway. Because of this, we only used the results in a one-step expansion. 

### 3.4. Pathway Network

**Figure 4:**
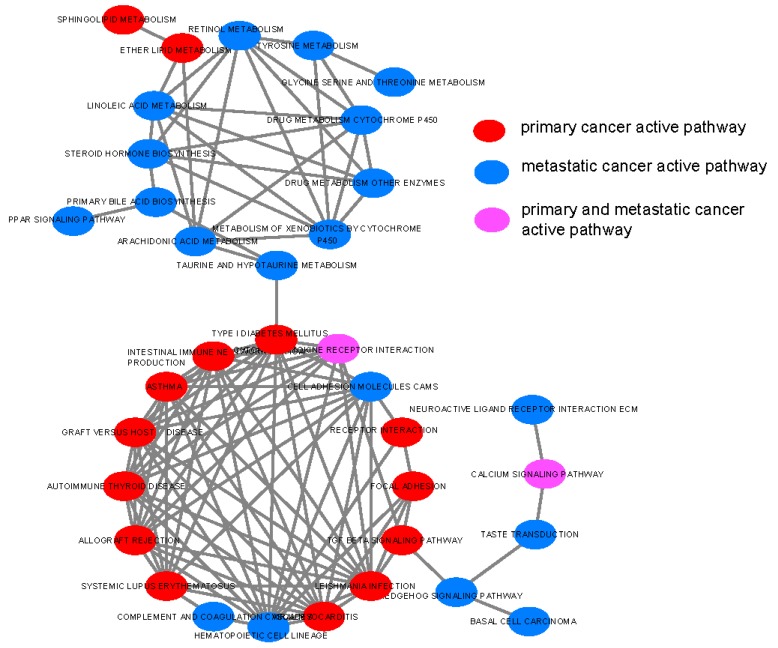
Pathway connections among 35 significantly differentially-active pathways in the general condition (general condition means in the raw KEGG pathways).

We found 109 pathway connections among 35 significant differentially-active pathways in the general condition without considering any disease status (Figure 4). Among these pathway connections, 86 pathway connections among 16 significant differentially-active pathways were in primary breast cancer (Supplemental Figure S2), and 96 pathway connections among 24 significant differentially-active pathways were in breast cancer liver metastasis (Supplemental Figure S3). The connections in the primary breast cancer or breast cancer liver metastasis were calculated based on the extended pathways separately. One hundred twelve pathway connections among 35 significant differential pathways were found to be in the cancer condition (primary cancer + metastatic cancer; Supplemental Figure S4).

As seen from these figures, there were two major clusters in the network. Primary cancer-related pathways were more likely connected with each other, while metastatic cancer-related pathways were more likely to connect with each other. The primary cancer-related pathways were frequently related to the immune system. The metastatic cancer-related pathways were frequently related to metabolism, such as linoleic acid metabolism. 

After comparing 86 pathway connections between the primary breast cancer and 96 pathway connections in the breast cancer liver metastasis, we found 70 common pathway connections. Next, we analyzed the 16 primary breast cancer-specific pathway connections (Supplemental Figure S5) and 26 breast cancer liver metastasis-specific pathway connections (Supplemental Figure S6). In Supplemental Figure S5, we find two hub pathways for primary breast cancer: *hematopoietic cell lineage* and *cell adhesion molecules cams*. In Supplemental Figure S6, we found a significant metabolism pathway clique and a hub pathway for the breast cancer liver metastasis: *TGF beta signaling*.

After comparing the pathway connections between the general condition and cancer condition, we found 103 common connections and nine cancer-specific connections (Supplemental Figure S7). Again, *TGF beta signaling* was found to be a hub pathway.

### 3.5. Alternatively-Activated Pathways

After getting the metastasis-related pathways, one important task was to explore the function of these pathways. In different conditions, such as primary cancer vs. metastatic cancer, the gene regulation patterns or topologies in pathways were different, and we refer to these active sub-pathways in this research as differentially-activated pathways. 

Taking the three significant metastasis-related pathways as an example, we recognized three distinctive types of alternatively-activated pathways. These three pathways were the *cytokine-cytokine receptor interaction*, *calcium signaling pathway,* and *TGF beta signaling* pathways. 

**Figure 5:**
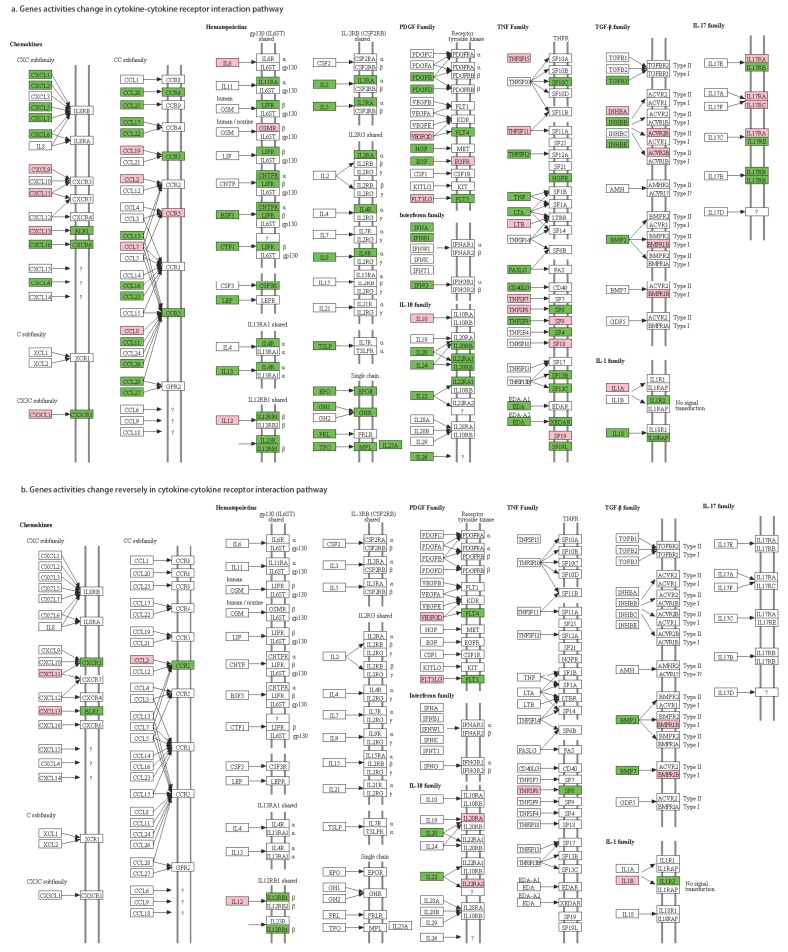
Gene activities in the *cytokine-cytokine receptor interaction* pathway.

In the *cytokine-cytokine receptor interaction* pathway, there was only one edge between each cytokine-cytokine receptor pair (Figure 5a). For each cytokine-cytokine receptor pair, the test results of gene-gene active alteration patterns are shown in Supplemental Table S4. For each gene-gene pair, there may be several significant active alteration patterns. We selected the pattern with the minimal *p*-value as its regulatory alteration pattern because the pattern with the minimal *p*-value should be the most possible pattern. Taking IL20-IL20RA (IL-10 family) as an example, patterns 00→10, 00→11, 01→10, 01→11 were all significant, but pattern 01→10 had the minimal *p*-value (0.00015), so we set this gene pair with the inverse pattern (both genes’ activity changes inversely). As a result, we found four gene-gene pairs with 01→10 patterns and nine gene-gene pairs with 10→01 patterns (Figure 5b).

The functions of these 13 cytokine-cytokine receptor pairs were further verified (Table 2). The results show that most of the cytokines or cytokine receptors (except IL12 and IL12RB1) were related to breast cancer or metastasis. This nicely verified our method for mining metastasis-related pathways and regulation pairs.

**Figure 6:**
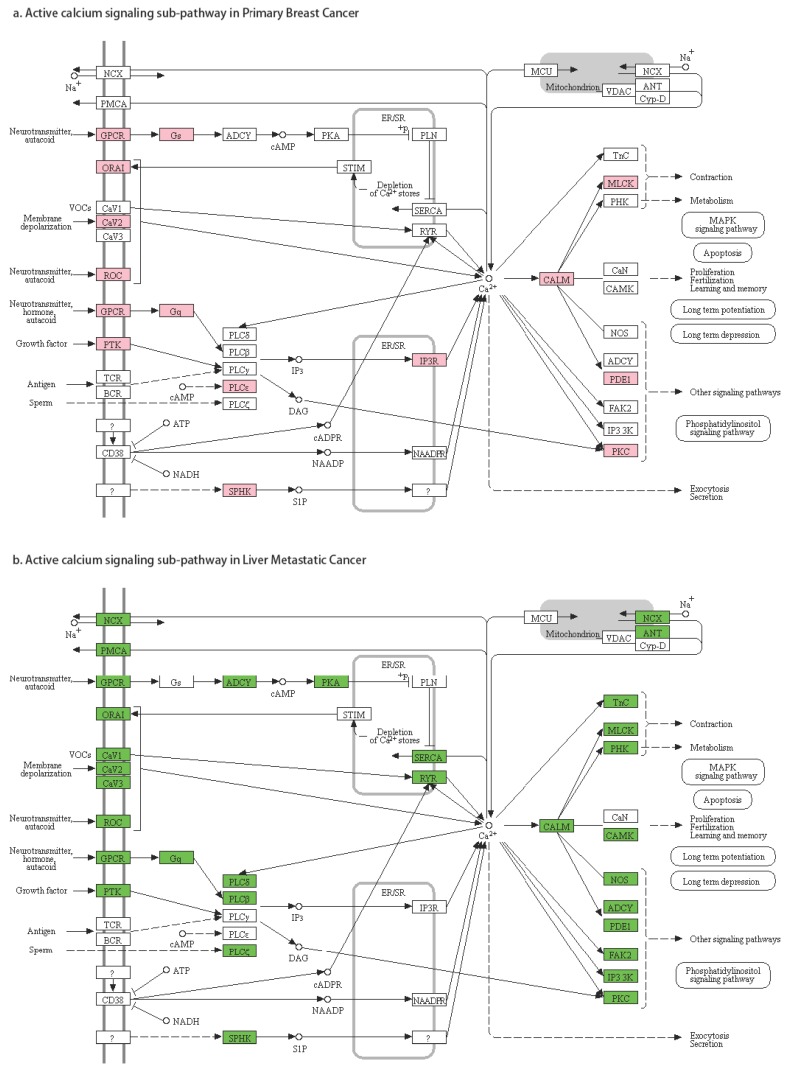
Gene activities in the *calcium signaling* pathway.

The second pattern of pathway regulation was that some sub-pathways in a pathway were active under one condition (primary breast cancer or breast cancer liver metastasis), but not in both. Identifying these alternatively-activated sub-pathways in the breast cancer liver metastases was our best interest. For example, in the *calcium signaling* pathway (Figure 6), we found that the sub-pathway TnC, PHK, CAMK, NOS, ADCY, FAK2, IP3-3K was only active in breast cancer liver metastasis.

**Figure 7:**
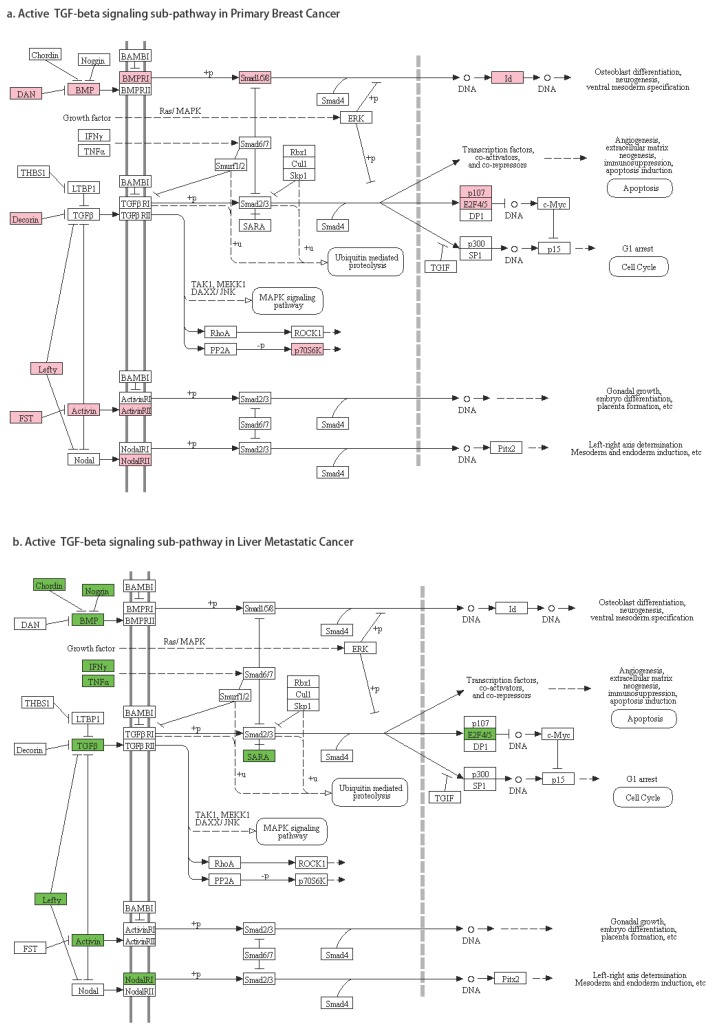
Gene activities in the *TGF beta signaling* pathway.

The third pattern of pathway regulation is that nodes were significantly active in both primary breast cancer and breast cancer liver metastasis, but the active genes in these nodes were different. Node CALM in *calcium signaling* represented any of these seven genes: CALM1, CALM2, CALM3, CALML3, CALML4, CALML5, and CALML6. We found that CALM2 and CALML5 were significantly active in primary breast cancer, while CALML3 and CALML6 were significantly active in breast cancer liver metastasis. Node BMP contained 11 different genes (GDF5, GDF6, GDF7, AMH, BMP2, BMP4, BMP5, BMP6, BMP7, BMP8A, BMP8B). BMP8B was significantly active in primary breast cancer, while BMP2, BMP4, BMP5, BMP6, BMP8B were significantly active in liver metastasis. Node E2F4/5 can be E2F4 or E2F5. E2F5 was significantly active in primary breast cancer, and E2F4 was significantly active in the metastasis. Therefore, a pathway may be active in both primary cancer and metastatic cancer, but their mechanisms could be different. In *TGF beta signaling pathway* (Figure 7), taking the sub-pathway from TGFB to E2F4/5 as an example, in primary breast cancer, gene DCN (node Decorin) was active, and it inhibited TGFB expression; however, RBL1 (node p107) and E2F5 were active (active sub-pathway DCN-TGFB-RBL1-E2F5); in breast to liver metastatic cancer, TGFB3 (node TGBF) was active, as well as E2F4 (active sub-pathway TGFB3-E2F4). 

## 4. Discussions

Instead of gene expression data, we used PMA information for each gene and divided genes into the active set and inactive set. Using an IGA-like strategy and integrating information from Bayesian networks, we identified several metastasis-related pathways. After putting the gene status into the significant pathways, we got three different types of alternatively-activated pathways. In most previous gene set enrichment analysis methods [32,33], only the gene expression data were used. To the best of our knowledge, our study is the first time that gene status information (PMA) was also used in pathway enrichment analysis. Compared to the gene expression data, the presence-absence calls information was more suitable when we say the gene was active or inactive instead of the gene expression value. For example, even genes that were not expressed generate signal values, usually low; random fluctuations in these low values can often produce large apparent fold changes. A gene also can be inactive even if the expression value was high when the background expression was also high [67,68]. Batch effects were the systematic error introduced when samples were processed in multiple batches. The batch effect is a common problem associated with microarray datasets and cannot be eliminated unless the whole study is done in a single batch. In this research, there were different platforms in the datasets, so PMA information instead of gene expression could reduce the differences between platforms and help in dealing with batch effects.

Molecular networks under the primary cancer and metastasis conditions are complex dynamic systems that demonstrate nonintuitive behaviors. Bayesian networks that infer probabilistic causal relationships between network components based on gene expression are widely used in cancer research [69,70]. Here, Bayesian networks constructed based on primary breast cancer and breast cancer liver metastasis conditions separately were used to expand the pathways. Comparing the condition-specific expanded pathways is more reasonable to understand the difference between them. After mapping the genes with different activity status into significant pathways, we identified three types of alternatively-activated pathways. Alternatively-activated pathways were widely researched in cancer and metastasis [71]. However, this alternatively-activated pathway concept has not been fully explored in the computational cancer system biology. This is the first time using microarray data to identify alternatively-activated pathways in breast cancer liver metastasis. 

In this study, we identified several metastasis-related pathways, such as the *cytokine-cytokine receptor interaction*, *calcium signaling*, and *TGF beta signaling* pathways, and these pathways have been proven to be related to breast cancer metastasis by previous research. Some cytokines (IL-1, IL-6, IL-11, TGFβ) stimulate, while others (IL-12, IL-18, IFNs) inhibit breast cancer proliferation and/or invasion [72]. In a few studies, multivariate analysis identified high serum IL-6 level as an independent adverse prognostic variable for progression-free and overall survival in metastatic breast cancer [73,74,75]. Calcium-activated chloride channel ANO1 was found to promote breast cancer progression [76]. Deregulation of calcium homeostasis and signaling is associated with mammary gland pathophysiology, and as such, calcium transporters, channels, and binding proteins represent potential drug targets in breast cancer [77]. The role of TGF-*beta* in breast cancer progression is ambiguous, as it was shown to display both tumor-suppressing and -promoting effects [78,79]. This is consistent with our results, which it is significant in both primary cancer and metastasis.

Three types of alternatively-activated pathways were identified in our research: active states of some gene-gene pairs were inversed; some sub-pathways were only active in primary cancer, or metastatic cancer; and some sub-pathways were alternatively-activated by different genes. In the TGF-β signaling pathway, E2F5 status was reported to improve the diagnosis of the malignancy of breast tumors [80], epithelial ovarian cancer [81], and hepatocellular carcinoma [82]. Higher E2F4 activity is predictive of worse survival [83]. In our study, we identified two different alternatively-activated TGF-β signaling pathways, and the E2F5-related sub-pathway is related to breast cancer, while the E2F4 related one is a risk for metastasis and potential worse survival. These results demonstrated the effectiveness of our alternatively-activated pathway methods and results. 

## 5. Conclusions 

Overall, in this study, we proposed an alternatively-activated pathway mining method based on microarray data and identified three types of alternatively-activated pathways between primary breast cancer and breast liver metastatic cancer. The results showed that our proposed method was valid and effective. In conclusion, our method may contribute to future mechanism study of breast cancer metastasis and eventually to the development of therapeutic drugs that may prevent cancer metastasis.

## Figures and Tables

**Table 1:** Number of significant KEGG pathways using different pathway resources.

Pathway Resource	Number of Significant Primary Cancer-Related Pathways	Number of Significant Metastatic Cancer-Related Pathways
Raw pathways	17	24
Expanded pathways by primary cancer Bayesian network 1 time	22	13
Expanded pathways by primary cancer Bayesian network 2 times	41	0
Expanded pathways by primary cancer Bayesian network 3 time	55	0
Expanded pathways by metastatic cancer Bayesian network 1 time	11	34
Expanded pathways by metastatic cancer Bayesian network 2 times	5	49
Expanded pathways by metastatic cancer Bayesian network 3 time	1	48

**Table 2:** Literature verification for the 13 cytokine-cytokine receptor pairs.

Type	Cytokine	Cytokine Receptor	Pattern	Function
Chemokines	CXCL11	CXCR3	10→01	CXCR3 is a molecular target in breast cancer metastasis [48,49]
	CXCL13	BLR1	10→01	CXCL13 is overexpressed in breast cancer patients [50,51]
	CCL2	CCR2	10→01	the CCL2-triggered chemokine cascade in macrophages promotes metastatic seeding of breast cancer cells, thereby amplifying the pathology already extant in the system [52] CCL2/CCR2 chemokine signaling coordinates the survival and motility of breast cancer cells with implications on the metastatic process [53]
PDG family	VEGFC	FLT4	10→01	activation of the VEGF-C/Flt-4 axis enhances mobility of cancer cells and contributes to the promotion of metastasis in animals [54] VEGF-C-VEGFR3/Flt4 axis regulates mammary tumor growth and metastasis in an autocrine manner [55] VEGF-C/D and Flt-4 may play an important role in the process of lymphatic metastasis of early-stage invasive cervical carcinoma through paracrine and autocrine mechanisms [56]
	FLT3LG	FLT3	10→01	FLT3-ligand administration inhibits liver metastases [57]
TNF family	TNFSF8	TNFRSF8	10→01	TNF inhibitor suppresses bone metastasis in a breast cancer cell line [58]
IL-1 family	IL1B	IL1R2	10→01	IL-1B is a potential biomarker for predicting breast cancer patients at increased risk for developing bone metastasis [59] IL-1 drives breast cancer growth and bone metastasis in vivo [60]
IL-10 family	IL20	IL20RA	01→10	IL-20 plays pivotal roles in the tumor progression of breast cancer; IL-20 may be a novel target in treating breast tumor-induced osteolysis [61]
	IL22	IL22RA2	01→10	IL-22 promotes epithelial cell transformation and breast tumorigenesis [62]
TGF-b family	BMP2	BMPR1B	01→10	Bone morphogenic proteins are related to driving breast cancer metastasis to bone [63] The BMP2/7 heterodimer inhibits the human breast cancer stem cell subpopulation and bone metastases’ formation [64] TGF-β activity is controlled by the expression of BMP7; BMP7 expression has been shown to be inversely proportional to the tumorigenicity and invasive behavior of MDA-MB-231 breast cancer cells [65] The presence of high levels of BMP7 expression in primary tumors has been strongly associated with accelerated bone metastasis, especially from ductal carcinomas [66]
	BMP7			
Hematopoietic	IL12A	IL12RB1	10→01	NA
	IL12B		10→01	

## Data Availability

The gene expression data were download from the GEO database. There were 153 samples in the primary breast cancer group [39] (GSE65194, Affymetrix Human Genome U133 Plus 2.0 Array) and 43 samples in the metastasis breast to liver cancer group [40,41] (GSE46141, 16 samples, GSE56493, 27 samples, Rosetta/Merck Human RSTA Custom Affymetrix 2.0 microarray). A total of 186 KEGG pathways and 676 REACTOME pathways were downloaded from MsigDB v6.0 [34].

## Supplementary Materials

The following are available online at https://www.mdpi.com/2073-4425/10/10/753/s1. Figure S1: Gene numbers in different raw and expended pathways, Figure S2: Pathway connections among 16 significant differentially-active pathways in the primary cancer condition (primary cancer condition means in the one-step primary cancer expanded KEGG pathways), Supplemental Figure S3: Pathway connections among 24 significant differentially-active pathways in the metastatic cancer condition (metastatic cancer condition means in the one-step metastatic cancer expanded KEGG pathways), Figure S4. Pathway connections among 35 significant differential pathways in the cancer condition (cancer condition means the combination of primary cancer condition and metastatic cancer condition), Figure S5: Primary cancer-specific active pathway connections, Figure S6: Metastatic cancer-specific active pathway connections, Figure S7: Cancer-specific active pathway connections, Table S1: Results of KEGG pathway analysis, Table S2: Comparison of active gene results from all samples and random samples, Table S3: Comparison of active pathway results from all samples and random samples, Table S4: The test results of gene-gene active alteration patterns in cytokine-cytokine receptor pathway.

## References

[1] Ferlay J., Soerjomataram I., Dikshit R., Eser S., Mathers C., Rebelo M., Parkin D.M., Forman D., Bray F. (2015). Cancer Incidence and Mortality Worldwide: Sources, Methods and Major Patterns in GLOBOCAN 2012. Int. J. Cancer.

[2] Mehlen P., Puisieux A. (2006). Metastasis: A question of life or death. Nat. Rev. Cancer.

[3] Karagiannis G.S., Goswami S., Jones J.G., Oktay M.H., Condeelis J.S. (2016). Signatures of breast cancer metastasis at a glance. J. Cell Sci..

[4] Van’t Veer L.J., Dai H., Van d.V.M.J., He Y.D., Hart A.A., Mao M., Peterse H.L., van der Kooy K., Marton M.J., Witteveen A.T. (2002). Gene expression profiling predicts clinical outcome of breast cancer. Nature.

[5] Van De Vijver M.J., He Y.D., Van‘t Veer L.J., Dai H., Hart A.A., Voskuil D.W., Schreiber G.J., Peterse J.L., Roberts C., Marton M.J. (2002). A Gene-Expression Signature as a Predictor of Survival in Breast Cancer. New Engl. J. Med..

[6] Bueno-de-Mesquita J.M., Linn S.C., Keijzer R., Wesseling J., Nuyten D.S., van Krimpen C., Meijers C., de Graaf P.W., Bos M.M., Hart A.A. (2009). Validation of 70-gene prognosis signature in node-negative breast cancer. Breast Cancer Res. Treat..

[7] Buyse M., Loi S., Veer L.V., Viale G., Delorenzi M., Glas A.M., D’Assignies M.S., Bergh J., Lidereau R., Ellis P. (2006). Validation and Clinical Utility of a 70-Gene Prognostic Signature for Women with Node-Negative Breast Cancer. J. Natl. Cancer Inst..

[8] Mook S., Schmidt M.K., Viale G., Pruneri G., Eekhout I., Floore A., Glas A.M., Bogaerts J., Cardoso F., Piccart-Gebhart M.J. (2009). The 70-gene prognosis-signature predicts disease outcome in breast cancer patients with 1-3 positive lymph nodes in an independent validation study. Breast Cancer Res Treat.

[9] Mook S., Schmidt M.K., Weigelt B., Kreike B., Eekhout I., van de Vijver M.J., Glas A.M., Floore A., Rutgers E.J., van’t Veer L.J. (2010). The 70-gene prognosis signature predicts early metastasis in breast cancer patients between 55 and 70 years of age. Ann. Oncol..

[10] Wittner B.S., Sgroi D.C., Ryan P.D., Bruinsma T.J., Glas A.M., Male A., Dahiya S., Habin K., Bernards R., Haber D.A. (2008). Analysis of the MammaPrint breast cancer assay in a predominantly postmenopausal cohort. Clin. Cancer Res..

[11] Curtis C. (2015). Genomic profiling of breast cancers. Curr. Opin. Obstet. Gynecol..

[12] Wang Y., Klijn J., Zhang Y., Sieuwerts A., Look M., Yang F., Talantov D., Timmermans M., Meijervangelder M., Yu J. (2005). Gene-expression profiles to predict distant metastasis of lymph-node-negative primary breast cancer. Lancet.

[13] Goldhirsch A., Glick J.H., Gelber R.D., Coates A.S., Senn H.-J. (2001). Meeting Highlights: International Consensus Panel on the Treatment of Primary Breast Cancer. J. Clin. Oncol..

[14] National Institutes of Health Consensus Development Panel (2001). National Institutes of Health Consensus Development Conference statement: Adjuvant therapy for breast cancer, 1–3 November 2000. J. Natl. Cancer Inst. Monogr..

[15] Sgroi D.C., Haber D.A., Ryan P.D., Ma X.-J., Erlander M.G. (2004). RE: A two-gene expression ratio predicts clinical outcome in breast cancer patients treated with tamoxifen. Cancer Cell.

[16] Paik S., Shak S., Tang G., Kim C., Baker J., Cronin M., Baehner F.L., Walker M.G., Watson D., Park T. (2004). A Multigene Assay to Predict Recurrence of Tamoxifen-Treated, Node-Negative Breast Cancer. N. Engl. J. Med..

[17] Landemaine T., Jackson A., Rucci N., Sin S., Abad B.M., Sierra A., Boudinet A., Ricevuto E., Briffod M., Cherel P. (2008). A Six-Gene Signature Predicting Breast Cancer Lung Metastasis. Cancer Res..

[18] Patsialou A., Wang Y., Lin J., Whitney K., Goswami S., A Kenny P., Condeelis J.S. (2012). Selective gene-expression profiling of migratory tumor cells in vivo predicts clinical outcome in breast cancer patients. Breast Cancer Res..

[19] Cejalvo J.M., De Dueñas E.M., Galvan P., García-Recio S., Gasión O.B., Paré L., Antolin S., Martinello R., Blancas I., Adamo B. (2017). Intrinsic Subtypes and Gene Expression Profiles in Primary and Metastatic Breast Cancer. Cancer Res..

[20] Hayashi N., Iwamoto T., Qi Y., Niikura N., Santarpia L., Yamauchi H., Nakamura S., Hortobagyi G.N., Pusztai L., Symmans W.F. (2017). Bone metastasis-related signaling pathways in breast cancers stratified by estrogen receptor status. J. Cancer.

[21] Bidwell B.N., Slaney C.Y., Withana N.P., Forster S., Cao Y., Loi S., Andrews D., Mikeska T., E Mangan N., A Samarajiwa S. (2012). Silencing of Irf7 pathways in breast cancer cells promotes bone metastasis through immune escape. Nat. Med..

[22] Sun M., Song C.-X., Huang H., Frankenberger C.A., Sankarasharma D., Gomes S., Chen P., Chen J., Chada K.K., He C. (2013). HMGA2/TET1/HOXA9 signaling pathway regulates breast cancer growth and metastasis. Proc. Natl. Acad. Sci. USA.

[23] Pai P., Rachagani S., Lakshmanan I., Macha M.A., Sheinin Y., Smith L.M., Ponnusamy M.P., Batra S.K. (2016). The canonical Wnt pathway regulates the metastasis-promoting mucin MUC4 in pancreatic ductal adenocarcinoma. Mol. Oncol..

[24] Wen X., Zhu J., Dong L., Chen Y. (2014). The role of c2orf68 and PI3K/Akt/mTOR pathway in human colorectal cancer. Med. Oncol..

[25] Papadatos-Pastos D., Rabbie R., Ross P., Sarker D. (2015). The role of the PI3K pathway in colorectal cancer. Crit. Rev. Oncol..

[26] Halaoui R., McCaffrey L. (2015). Rewiring cell polarity signaling in cancer. Oncogene.

[27] Lee M.J., Ye A.S., Gardino A.K., Heijink A.M., Sorger P.K., MacBeath G., Yaffe M.B. (2012). Sequential application of anticancer drugs enhances cell death by rewiring apoptotic signaling networks. Cell.

[28] Lopez-Bergami P., Huang C., Goydos J.S., Yip D., Bar-Eli M., Herlyn M., Smalley K.S.M., Mahale A., Eroshkin A., Aaronson S. (2007). Re-wired ERK-JNK signaling pathways in melanoma. Cancer Cell.

[29] Cotton T.B., Nguyen H.H., Said J.I., Ouyang Z., Zhang J., Song M. (2015). Discerning mechanistically rewired biological pathways by cumulative interaction heterogeneity statistics. Sci. Rep..

[30] Li J., Wang L., Xu L., Zhang R., Huang M., Wang K., Xu J., Lv H., Shang Z., Zhang M. (2012). DBGSA: A novel method of distance-based gene set analysis. J. Hum. Genet..

[31] Minn A.J., Bevilacqua E., Yun J., Rosner M.R. (2012). Identification of novel metastasis suppressor signaling pathways for breast cancer. Cell Cycle.

[32] Huang da W., Sherman B.T., Lempicki R.A. (2009). Systematic and integrative analysis of large gene lists using DAVID bioinformatics resources. Nat. Protoc..

[33] Huang da W., Sherman B.T., Lempicki R.A. (2009). Bioinformatics enrichment tools: Paths toward the comprehensive functional analysis of large gene lists. Nucleic Acids Res..

[34] Subramanian A., Tamayo P., Mootha V.K., Mukherjee S., Ebert B.L., Gillette M.A., Paulovich A., Pomeroy S.L., Golub T.R., Lander E.S. (2005). Gene set enrichment analysis: A knowledge-based approach for interpreting genome-wide expression profiles. Proc. Natl. Acad. Sci. USA.

[35] Khatri P., Sirota M., Butte A.J. (2012). Ten Years of Pathway Analysis: Current Approaches and Outstanding Challenges. PLoS Comput. Boil..

[36] Mitrea C., Taghavi Z., Bokanizad B., Hanoudi S., Tagett R., Donato M., Voichita C., Draghici S. (2013). Methods and approaches in the topology-based analysis of biological pathways. Front. Physiol..

[37] Wang W., Eddy R., Condeelis J. (2007). The cofilin pathway in breast cancer invasion and metastasis. Nat. Rev. Cancer.

[38] Kang Y., He W., Tulley S., Gupta G.P., Serganova I., Chen C.-R., Manova-Todorova K., Blasberg R., Gerald W.L., Massagué J. (2005). Breast cancer bone metastasis mediated by the Smad tumor suppressor pathway. Proc. Natl. Acad. Sci. USA.

[39] Maire V., Nemati F., Richardson M., Vincent-Salomon A., Tesson B., Rigaill G., Gravier E., Marty-Prouvost B., de Koning L., Lang G. (2013). Polo-like kinase 1: A potential therapeutic option in combination with conventional chemotherapy for the management of patients with triple-negative breast cancer. Cancer Res..

[40] Kimbung S., Kovacs A., Bendahl P.O., Malmstrom P., Ferno M., Hatschek T., Hedenfalk I. (2014). Claudin-2 is an independent negative prognostic factor in breast cancer and specifically predicts early liver recurrences. Mol. Oncol..

[41] Tobin N.P., Harrell J.C., Lovrot J., Brage S.E., Stolt M.F., Carlsson L., Einbeigi Z., Linderholm B., Loman N., Malmberg M. (2015). Molecular subtype and tumor characteristics of breast cancer metastases as assessed by gene expression significantly influence patient post-relapse survival. Ann. Oncol..

[42] Cheng F., Jia P., Wang Q., Lin C.-C., Li W.-H., Zhao Z. (2014). Studying Tumorigenesis through Network Evolution and Somatic Mutational Perturbations in the Cancer Interactome. Mol. Boil. Evol..

[43] Cheng F., Liu C., Shen B., Zhao Z. (2016). Investigating cellular network heterogeneity and modularity in cancer: A network entropy and unbalanced motif approach. BMC Syst. Boil..

[44] Chickering D.M. (2002). Optimal Structure Identification with Greedy Search. J. Mach. Learn. Res..

[45] Ramsey J.D. (2015). Scaling up Greedy Causal Search for Continuous Variables. arXiv.

[46] Benjamini Y., Hochberg Y. (1995). Controlling the false discovery rate—A practical and powerful approach to multiple testing. J. Royal Stat. Soc. Ser. B Methodol..

[47] Shannon P., Markiel A., Ozier O., Baliga N.S., Wang J.T., Ramage D., Amin N., Schwikowski B., Ideker T. (2003). Cytoscape: A Software Environment for Integrated Models of Biomolecular Interaction Networks. Genome Res..

[48] Zhu G., Yan H.H., Pang Y., Jian J., Achyut B.R., Liang X., Weiss J.M., Wiltrout R.H., Hollander M.C., Yang L. (2015). CXCR3 as a molecular target in breast cancer metastasis: Inhibition of tumor cell migration and promotion of host anti-tumor immunity. Oncotarget.

[49] Ma X., Norsworthy K., Kundu N., Rodgers W.H., Gimotty P.A., Goloubeva O., Lipsky M., Li Y., Holt D., Fulton A. (2009). CXCR3 expression is associated with poor survival in breast cancer and promotes metastasis in a murine model. Mol. Cancer Ther..

[50] Panse J., Friedrichs K., Marx A., Hildebrandt Y., Luetkens T., Bartels K., Horn C., Stahl T., Cao Y., Milde-Langosch K. (2008). Chemokine CXCL13 is overexpressed in the tumour tissue and in the peripheral blood of breast cancer patients. Br. J. Cancer.

[51] Chen L., Huang Z., Yao G., Lyu X., Li J., Hu X., Cai Y., Li W., Li X., Ye C. (2015). The expression of CXCL13 and its relation to unfavorable clinical characteristics in young breast cancer. J. Transl. Med..

[52] Kitamura T., Qian B.-Z., Soong D., Cassetta L., Noy R., Sugano G., Kato Y., Li J., Pollard J.W. (2015). CCL2-induced chemokine cascade promotes breast cancer metastasis by enhancing retention of metastasis-associated macrophages. J. Cell Boil..

[53] Bin Fang W., Jokar I., Zou A., Lambert D., Dendukuri P., Cheng N. (2012). CCL2/CCR2 Chemokine Signaling Coordinates Survival and Motility of Breast Cancer Cells through Smad3 Protein- and p42/44 Mitogen-activated Protein Kinase (MAPK)-dependent Mechanisms. J. Boil. Chem..

[54] Su J.-L., Yang P.-C., Shih J.-Y., Yang C.-Y., Wei L.-H., Hsieh C.-Y., Chou C.-H., Jeng Y.-M., Wang M.-Y., Chang K.-J. (2006). The VEGF-C/Flt-4 axis promotes invasion and metastasis of cancer cells. Cancer Cell.

[55] Varney M.L., Singh R.K. (2015). VEGF-C-VEGFR3/Flt4 axis regulates mammary tumor growth and metastasis in an autocrine manner. Am. J. Cancer Res..

[56] Yu H., Zhang S., Zhang R., Zhang L. (2009). The role of VEGF-C/D and Flt-4 in the lymphatic metastasis of early-stage invasive cervical carcinoma. J. Exp. Clin. Cancer Res..

[57] Péron J.M., Esche C., Subbotin V.M., Maliszewski C., Lotze M.T., Shurin M.R. (1998). FLT3-ligand administration inhibits liver metastases: Role of NK cells. J. Immunol..

[58] Hamaguchi T., Wakabayashi H., Matsumine A., Sudo A., Uchida A. (2011). TNF inhibitor suppresses bone metastasis in a breast cancer cell line. Biochem. Biophys. Res. Commun..

[59] Nutter F., Holen I., Brown H.K., Cross S.S., Evans C.A., Walker M., E Coleman R., A Westbrook J., Selby P.J., E Brown J. (2014). Different molecular profiles are associated with breast cancer cell homing compared with colonisation of bone: Evidence using a novel bone-seeking cell line. Endocr.-Relat. Cancer.

[60] Holen I., Lefley D.V., Francis S.E., Rennicks S., Bradbury S., Coleman R.E., Ottewell P. (2016). IL-1 drives breast cancer growth and bone metastasis in vivo. Oncotarget.

[61] Hsu Y.-H., Hsing C.-H., Li C.-F., Chan C.-H., Chang M.-C., Yan J.-J., Chang M.-S. (2012). Anti–IL-20 Monoclonal Antibody Suppresses Breast Cancer Progression and Bone Osteolysis in Murine Models. J. Immunol..

[62] Kim K., Kim G., Kim J.-Y., Yun H.J., Lim S.-C., Choi H.S. (2014). Interleukin-22 promotes epithelial cell transformation and breast tumorigenesis via MAP3K8 activation. Carcinog..

[63] Ottewell P.D., O’Donnell L., Holen I. (2015). Molecular alterations that drive breast cancer metastasis to bone. BoneKEy Rep..

[64] Buijs J.T., van der Horst G., van den Hoogen C., Cheung H., de Rooij B., Kroon J., Petersen M., van Overveld P.G., Pelger R.C., van der Pluijm G. (2012). The BMP2/7 heterodimer inhibits the human breast cancer stem cell subpopulation and bone metastases formation. Oncogene.

[65] Buijs J.T., Henriquez N.V., Van Overveld P.G., Que I., Schwaninger R., Rentsch C., Dijke P.T., Driouch K., Lidereau R., Vukicevic S. (2007). Bone Morphogenetic Protein 7 in the Development and Treatment of Bone Metastases from Breast Cancer. Cancer Res..

[66] Buijs J.T., Petersen M., Van Der Horst G., Van Der Pluijm G. (2010). Bone morphogenetic proteins and its receptors; therapeutic targets in cancer progression and bone metastasis?. Curr. Pharm. Des..

[67] McClintick J.N., Edenberg H.J. (2006). Effects of filtering by Present call on analysis of microarray experiments. BMC Bioinform..

[68] Strauß L., Ruffing U., Abdulla S., Alabi A., Akulenko R., Garrine M., Germann A., Grobusch M.P., Helms V., Herrmann M. (2016). Detecting Staphylococcus aureus Virulence and Resistance Genes: A Comparison of Whole-Genome Sequencing and DNA Microarray Technology. J. Clin. Microbiol..

[69] Gendelman R., Xing H., Mirzoeva O.K., Sarde P., Curtis C., Feiler H.S., McDonagh P., Gray J.W., Khalil I., Korn W.M. (2017). Bayesian Network Inference Modeling Identifies TRIB1 as a Novel Regulator of Cell-Cycle Progression and Survival in Cancer Cells. Cancer Res..

[70] Cai Z.-Q., Guo P., Si S.-B., Geng Z.-M., Chen C., Cong L.-L. (2017). Analysis of prognostic factors for survival after surgery for gallbladder cancer based on a Bayesian network. Sci. Rep..

[71] Cantor J.R., Sabatini D.M. (2012). Cancer cell metabolism: One hallmark, many faces. Cancer Discov..

[72] Chen W., Qin Y., Liu S. (2018). Cytokines, breast cancer stem cells (BCSCs) and chemoresistance. Clin. Transl. Med..

[73] Zhang G.J., Adachi I. (1999). Serum interleukin-6 levels correlate to tumor progression and prognosis in metastatic breast carcinoma. Anticancer. Res..

[74] Bachelot T., Ray-Coquard I., Menetrier-Caux C., Rastkha M., Duc A., Blay J.-Y. (2003). Prognostic value of serum levels of interleukin 6 and of serum and plasma levels of vascular endothelial growth factor in hormone-refractory metastatic breast cancer patients. Br. J. Cancer.

[75] Bozcuk H., Uslu G., Samur M., Yıldız M., Ozben T., Ozdogan M., Artaç M., Altunbas H., Akan I., Savas B. (2004). Tumour necrosis factor-alpha, interleukin-6, and fasting serum insulin correlate with clinical outcome in metastatic breast cancer patients treated with chemotherapy. Cytokine.

[76] Britschgi A., Bill A., Brinkhaus H., Rothwell C., Clay I., Duss S., Rebhan M., Raman P., Guy C.T., Wetzel K. (2013). Calcium-activated chloride channel ANO1 promotes breast cancer progression by activating EGFR and CAMK signaling. Proc. Natl. Acad. Sci. USA.

[77] Lee W.J., Monteith G.R., Roberts-Thomson S.J. (2006). Calcium transport and signaling in the mammary gland: Targets for breast cancer. Biochim. Biophys. Acta.

[78] Wakefield L.M., Roberts A.B. (2002). TGF-beta signaling: Positive and negative effects on tumorigenesis. Curr. Opin. Genet. Dev..

[79] Buck M.B., Knabbe C. (2006). TGF-Beta Signaling in Breast Cancer. Ann. N. Y. Acad. Sci..

[80] Polanowska J., Le Cam L., Orsetti B., Vallès H., Fabbrizio E., Fajas L., Taviaux S., Theillet C., Sardet C. (2000). Human E2F5 gene is oncogenic in primary rodent cells and is amplified in human breast tumors. Genes Chromosom. Cancer.

[81] Kothandaraman N., Bajic V.B., Brendan P.N., Huak C.Y., Keow P.B., Razvi K., Salto-Tellez M., Choolani M. (2010). E2F5 status significantly improves malignancy diagnosis of epithelial ovarian cancer. BMC Cancer.

[82] Jiang Y., Yim S.-H., Xu H.-D., Jung S.-H., Yang S.Y., Hu H.-J., Jung C.-K., Chung Y.-J. (2011). A potential oncogenic role of the commonly observed E2F5 overexpression in hepatocellular carcinoma. World J. Gastroenterol..

[83] Khaleel S.S., Andrews E.H., Ung M., DiRenzo J., Cheng C. (2014). E2F4 regulatory program predicts patient survival prognosis in breast cancer. Breast Cancer Res..

