# Supplementary material for: Identification of Alternatively-Activated Pathways between Primary Breast Cancer and Liver Metastatic Cancer Using Microarray Data
Source: Genes (Basel). 2019 Sep 25;10(10):753. doi: 10.3390/genes10100753 (PMC6826985; doi:10.3390/genes10100753)
Supplement: Supplementary file 1 [file genes-10-00753-s001.zip › figures and tables final/table 2 Literature verification for the 13 cytokine-cytokine receptor pairs.docx]

**Table 2.** Literature verification for the 13 cytokine-cytokine receptor pairs.

| **Type** | **Cytokine** | **Cytokine Receptor** | **Pattern** | **Function** |
| --- | --- | --- | --- | --- |
| Chemokines | CXCL11 | CXCR3 | 10→01 | CXCR3 is a molecular target in breast cancer metastasis [48,49] |
|  | CXCL13 | BLR1 | 10→01 | CXCL13 is overexpressed in breast cancer patients [50,51] |
|  | CCL2 | CCR2 | 10→01 | the CCL2-triggered chemokine cascade in macrophages promotes metastatic seeding of breast cancer cells, thereby amplifying the pathology already extant in the system [529]  CCL2/CCR2 chemokine signaling coordinates the survival and motility of breast cancer cells with implications on the metastatic process [53] |
| PDG family | VEGFC | FLT4 | 10→01 | activation of the VEGF-C/Flt-4 axis enhances mobility of cancer cells and contributes to the promotion of metastasis  in animals [54]  VEGF-C-VEGFR3/Flt4 axis regulates mammary tumor growth and metastasis in an autocrine manner [55]  VEGF-C/D and Flt-4 may play an important role in the process of lymphatic metastasis of early-stage invasive cervical carcinoma through paracrine and autocrine mechanisms [56] |
|  | FLT3LG | FLT3 | 10→01 | FLT3-ligand administration inhibits liver metastases [57] |
| TNF family | TNFSF8 | TNFRSF8 | 10→01 | TNF inhibitor suppresses bone metastasis in a breast cancer cell line [58] |
| IL-1 family | IL1B | IL1R2 | 10→01 | IL-1B is a potential biomarker for predicting breast cancer patients at increased risk for developing bone metastasis [59]  IL-1 drives breast cancer growth and bone metastasis in vivo [60] |
| IL-10 family | IL20 | IL20RA | 01→10 | IL-20 plays pivotal roles in the tumor progression of breast cancer; IL-20 may be a novel target in treating breast tumor-induced osteolysis [61] |
|  | IL22 | IL22RA2 | 01→10 | IL-22 promotes epithelial cell transformation and breast tumorigenesis [62] |
| TGF-b family | BMP2 | BMPR1B | 01→10 | Bone morphogenic proteins are related to driving breast cancer metastasis to bone [63]  The BMP2/7 heterodimer inhibits the human breast cancer stem cell subpopulation and bone metastases’ formation [64]  TGF-β activity is controlled by the expression of BMP7; BMP7 expression has been shown to be inversely proportional to the tumorigenicity and invasive behavior of MDA-MB-231 breast cancer cells [65]  The presence of high levels of BMP7 expression in primary tumors has been strongly associated with accelerated bone metastasis, especially from ductal carcinomas [66] |
|  | BMP7 |  |  |  |
| Hematopoietic | IL12A | IL12RB1 | 10→01 | NA |
|  | IL12B |  | 10→01 |  |
