# Supplementary material for: Identification of Alternatively-Activated Pathways between Primary Breast Cancer and Liver Metastatic Cancer Using Microarray Data
Source: Genes (Basel). 2019 Sep 25;10(10):753. doi: 10.3390/genes10100753 (PMC6826985; doi:10.3390/genes10100753)

a. Active TGF-beta signaling sub-pathway in Primary Breast Cancer

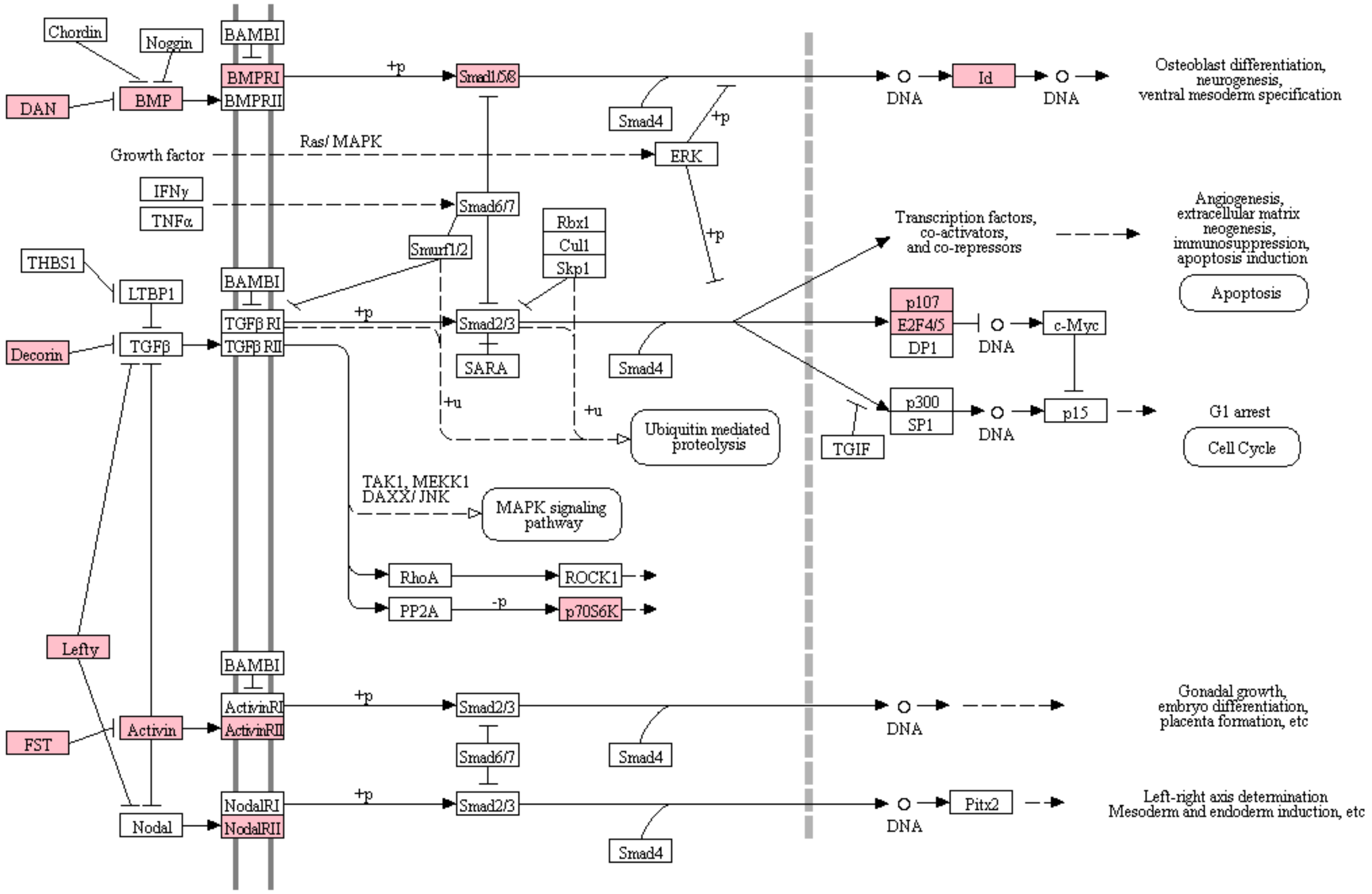

b. Active TGF-beta signaling sub-pathway in Liver Metastatic Cancer

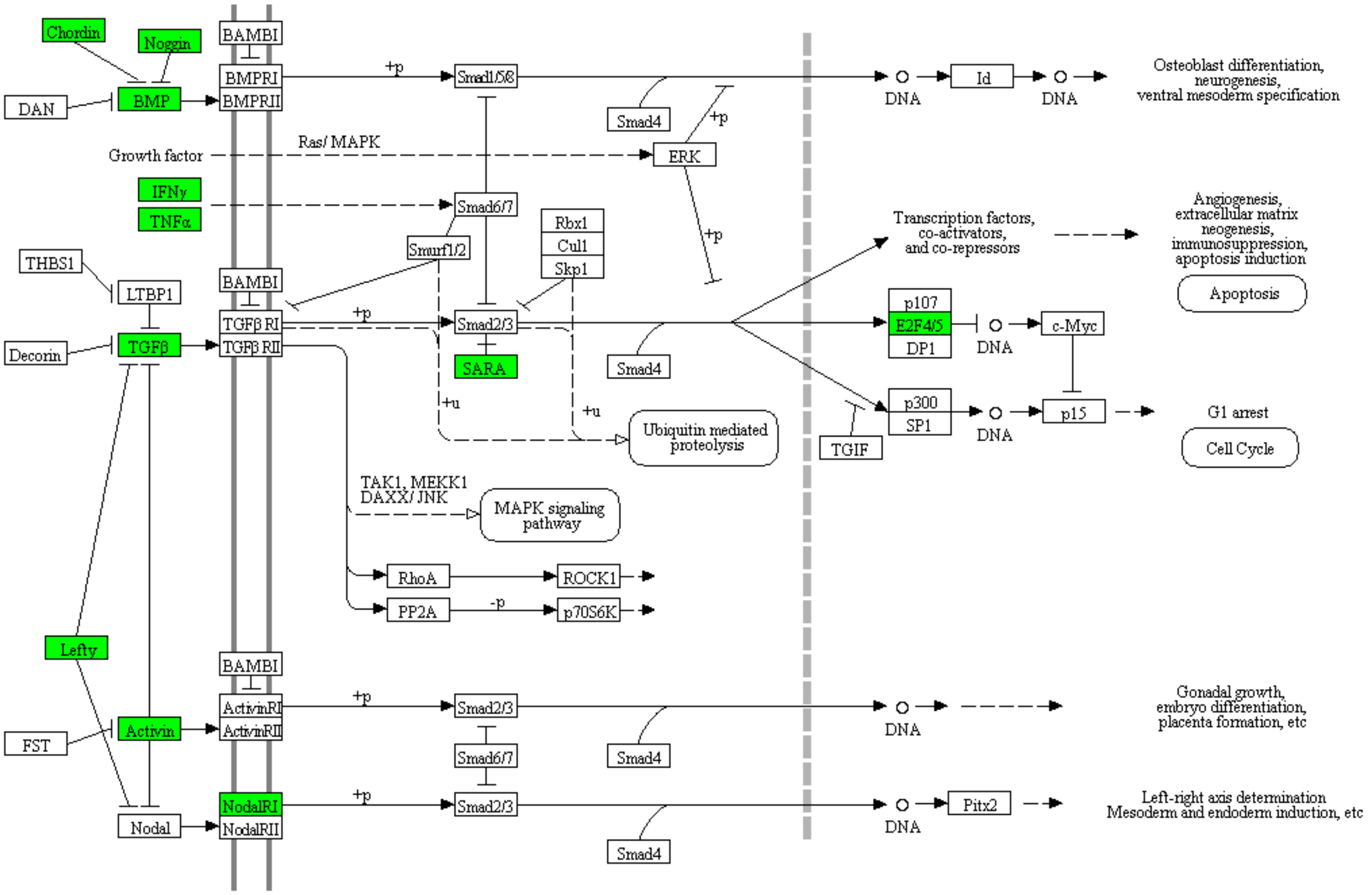

Supplement: Supplementary file 1 [file genes-10-00753-s001.zip › figures and tables final/figure 7.pdf]
