# Supplementary material for: Identification of Alternatively-Activated Pathways between Primary Breast Cancer and Liver Metastatic Cancer Using Microarray Data
Source: Genes (Basel). 2019 Sep 25;10(10):753. doi: 10.3390/genes10100753 (PMC6826985; doi:10.3390/genes10100753)
Supplement: Supplementary file 1 [file genes-10-00753-s001.zip › figures and tables final/supplenmentary table s3.docx]

**Supplemental table 3.** comparison of active pathway results from all samples and random samples.

| **random round** | **# of primary cancer active pathways in** | | | |  | **# of metastatic cancer active pathways in** | | | | |  |
| --- | --- | --- | --- | --- | --- | --- | --- | --- | --- | --- | --- |
|  | **All samples** | **Random samples** | **Common** | **Pearson correlation coefficient of p values from all samples and random samples** |  | | **All samples** | **Random samples** | **Common** | **Pearson correlation coefficient of p values from all samples and random samples** | |
| 1 | 17 | 13 | 12 | 0.89396 |  | | 23 | 22 | 19 | 0.9487505 | |
| 2 | 17 | 13 | 10 | 0.90524 |  | | 23 | 25 | 22 | 0.9678742 | |
| 3 | 17 | 11 | 10 | 0.89619 |  | | 23 | 22 | 20 | 0.9570949 | |
| 4 | 17 | 12 | 11 | 0.90096 |  | | 23 | 23 | 22 | 0.9627989 | |
| 5 | 17 | 11 | 10 | 0.89929 |  | | 23 | 23 | 21 | 0.9692735 | |
| 6 | 17 | 11 | 10 | 0.90826 |  | | 23 | 23 | 21 | 0.9576242 | |
| 7 | 17 | 12 | 11 | 0.92196 |  | | 23 | 24 | 21 | 0.963518 | |
| 8 | 17 | 12 | 12 | 0.90223 |  | | 23 | 23 | 23 | 0.9692031 | |
| 9 | 17 | 14 | 11 | 0.9138 |  | | 23 | 24 | 22 | 0.9673447 | |
| 10 | 17 | 10 | 9 | 0.89327 |  | | 23 | 24 | 22 | 0.9691399 | |
| average | 17 | 11.9 | 10.6 | 0.903516 |  | | 23 | 23.3 | 21.3 | 0.96326219 | |
