# Supplementary material for: Identification of Alternatively-Activated Pathways between Primary Breast Cancer and Liver Metastatic Cancer Using Microarray Data
Source: Genes (Basel). 2019 Sep 25;10(10):753. doi: 10.3390/genes10100753 (PMC6826985; doi:10.3390/genes10100753)
Supplement: Supplementary file 1 [file genes-10-00753-s001.zip › figures and tables final/supplenmentary table s2.docx]

**Supplemental table 2.** comparison of active gene results from all samples and random samples.

| **random round** | **# of primary cancer active genes** | | | |  | **# of metastatic cancer active genes** | | | |
| --- | --- | --- | --- | --- | --- | --- | --- | --- | --- |
|  | **All samples** | **random samples** | **common** | **Common/random samples** |  | **All samples** | **random samples** | **common** | **Common/random samples** |
| 1 | 1481 | 1219 | 1163 | 0.9541 |  | 4523 | 3706 | 3679 | 0.9927 |
| 2 | 1481 | 1155 | 1132 | 0.9801 |  | 4523 | 3916 | 3867 | 0.9875 |
| 3 | 1481 | 1173 | 1133 | 0.9659 |  | 4523 | 3865 | 3805 | 0.9845 |
| 4 | 1481 | 1168 | 1136 | 0.9726 |  | 4523 | 3931 | 3869 | 0.9842 |
| 5 | 1481 | 1162 | 1131 | 0.9733 |  | 4523 | 3892 | 3846 | 0.9882 |
| 6 | 1481 | 1150 | 1125 | 0.9783 |  | 4523 | 3953 | 3870 | 0.9790 |
| 7 | 1481 | 1148 | 1121 | 0.9765 |  | 4523 | 3888 | 3844 | 0.9887 |
| 8 | 1481 | 1143 | 1122 | 0.9816 |  | 4523 | 3967 | 3893 | 0.9813 |
| 9 | 1481 | 1166 | 1139 | 0.9768 |  | 4523 | 3892 | 3841 | 0.9869 |
| 10 | 1481 | 1150 | 1129 | 0.9817 |  | 4523 | 3946 | 3885 | 0.9845 |
| average | 1481 | 1163.4 | 1131.1 | 0.9741 |  | 4523 | 3895.6 | 3839.9 | 0.9858 |
