# Supplementary material for: Identification of Alternatively-Activated Pathways between Primary Breast Cancer and Liver Metastatic Cancer Using Microarray Data
Source: Genes (Basel). 2019 Sep 25;10(10):753. doi: 10.3390/genes10100753 (PMC6826985; doi:10.3390/genes10100753)
Supplement: Supplementary file 1 [file genes-10-00753-s001.zip › figures and tables final/figure 2 regulation alteration pattern_new.pdf]

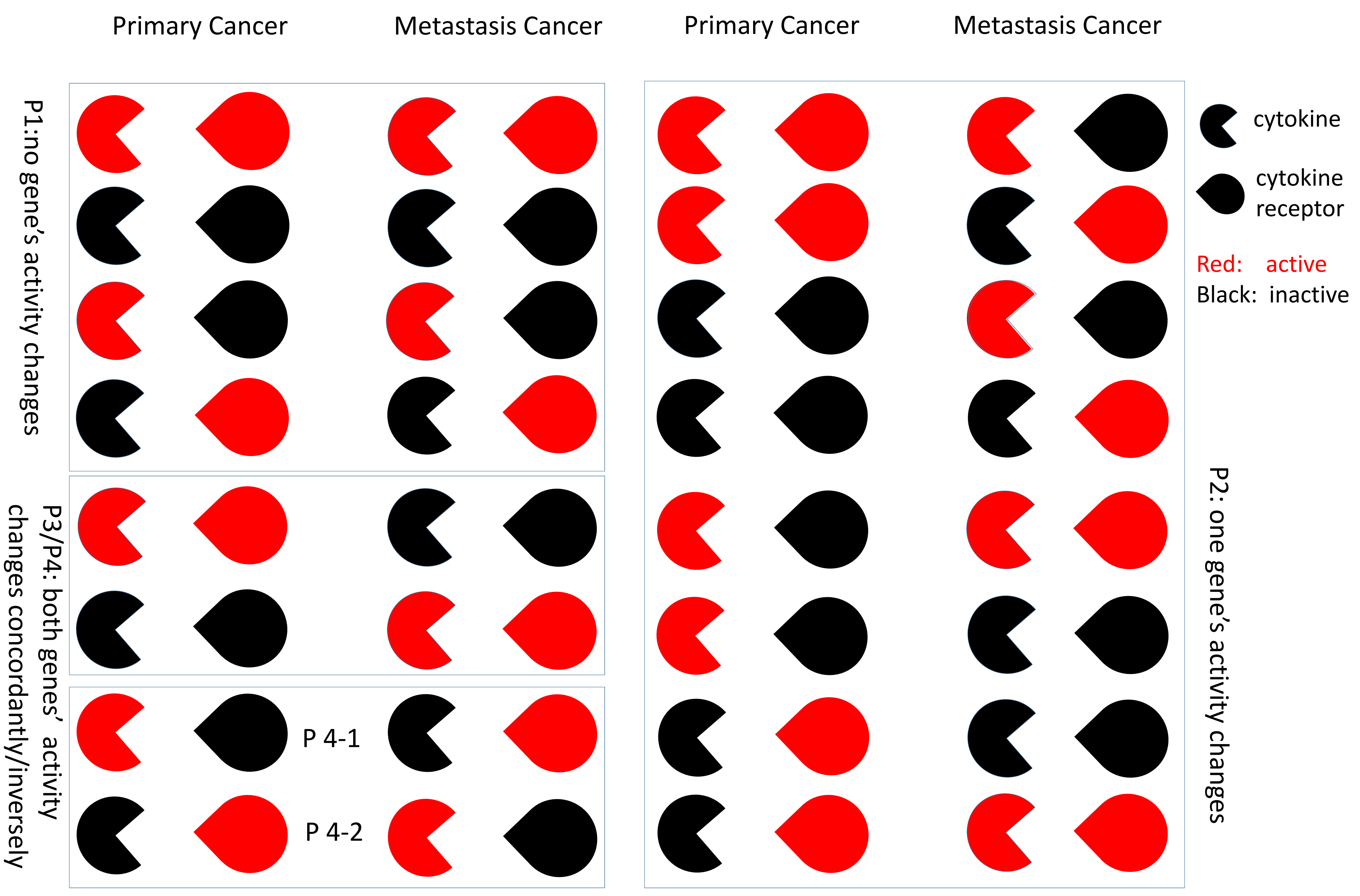
Primary Cancer
Metastasis Cancer
Primary Cancer
Metastasis Cancer
P1:no gene's activity changes
P3/P4: both genes' activity
changes concordantly/inversely
P 4-1
P 4-2
P2: one gene's activity changes
cytokine
cytokine
receptor
Red: active
Black: inactive
