# Supplementary material for: Identification of Alternatively-Activated Pathways between Primary Breast Cancer and Liver Metastatic Cancer Using Microarray Data
Source: Genes (Basel). 2019 Sep 25;10(10):753. doi: 10.3390/genes10100753 (PMC6826985; doi:10.3390/genes10100753)
Supplement: Supplementary file 1 [file genes-10-00753-s001.zip › figures and tables final/table 1 significant pathway with expanded netwrok.docx]

**Table 1.** number of significant KEGG pathways using different pathway resource.

| **Pathway resource** | **Number of significant primary cancer related pathways** | **Number of significant metastatic cancer related pathways** |
| --- | --- | --- |
| Raw pathways | 17 | 24 |
| Expanded pathways by primary cancer Bayesian network 1 time | 22 | 13 |
| Expanded pathways by primary cancer Bayesian network 2 times | 41 | 0 |
| Expanded pathways by primary cancer Bayesian network 3 time | 55 | 0 |
| Expanded pathways by metastatic cancer Bayesian network 1 time | 11 | 34 |
| Expanded pathways by metastatic cancer Bayesian network 2 times | 5 | 49 |
| Expanded pathways by metastatic cancer Bayesian network 3 time | 1 | 48 |
