# Supplementary figures and images for: Identification of Alternatively-Activated Pathways between Primary Breast Cancer and Liver Metastatic Cancer Using Microarray Data
Source: Genes (Basel). 2019 Sep 25;10(10):753. doi: 10.3390/genes10100753 (PMC6826985; doi:10.3390/genes10100753)

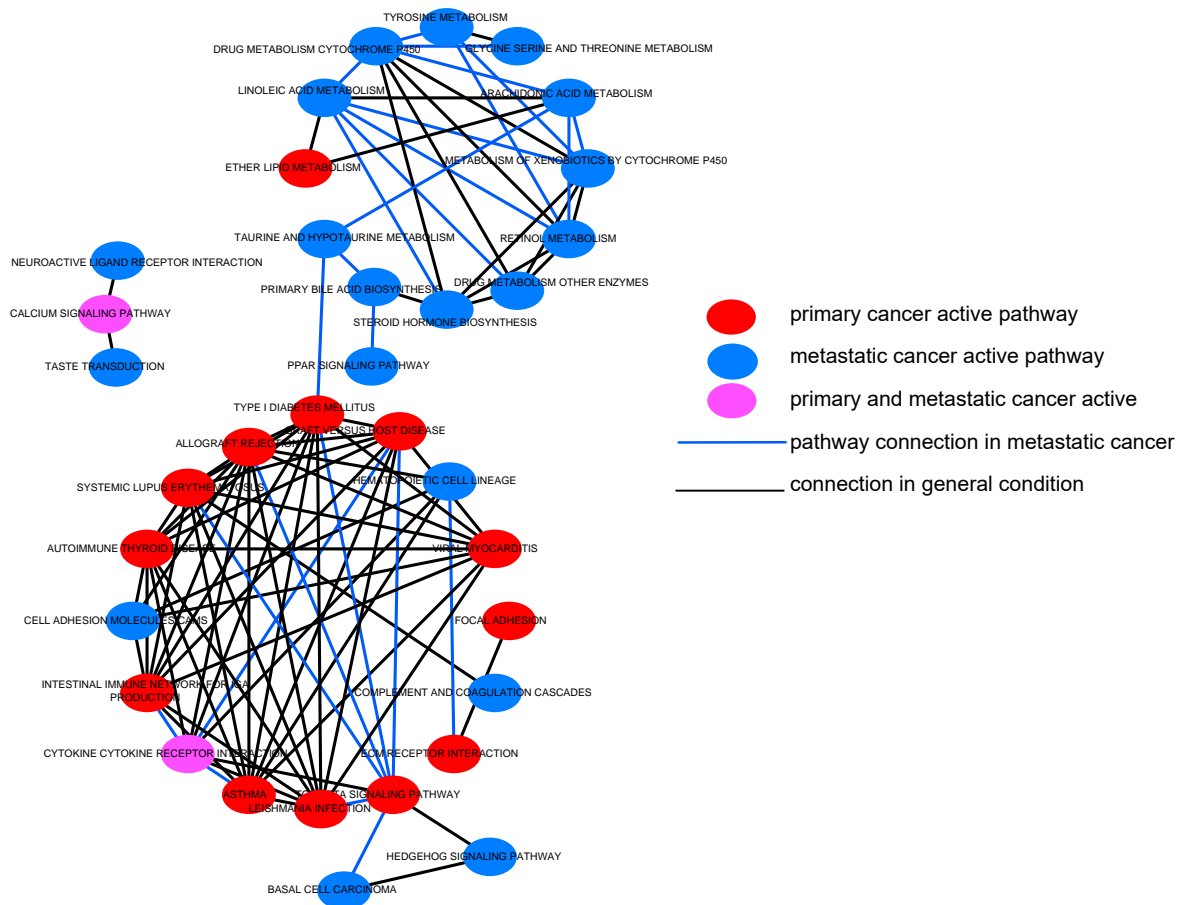

Supplement: Supplementary file 1 [file genes-10-00753-s001.zip › figures and tables final/supplemental figure s3 pathway_common_and_seprate_96 metastasis.pdf]

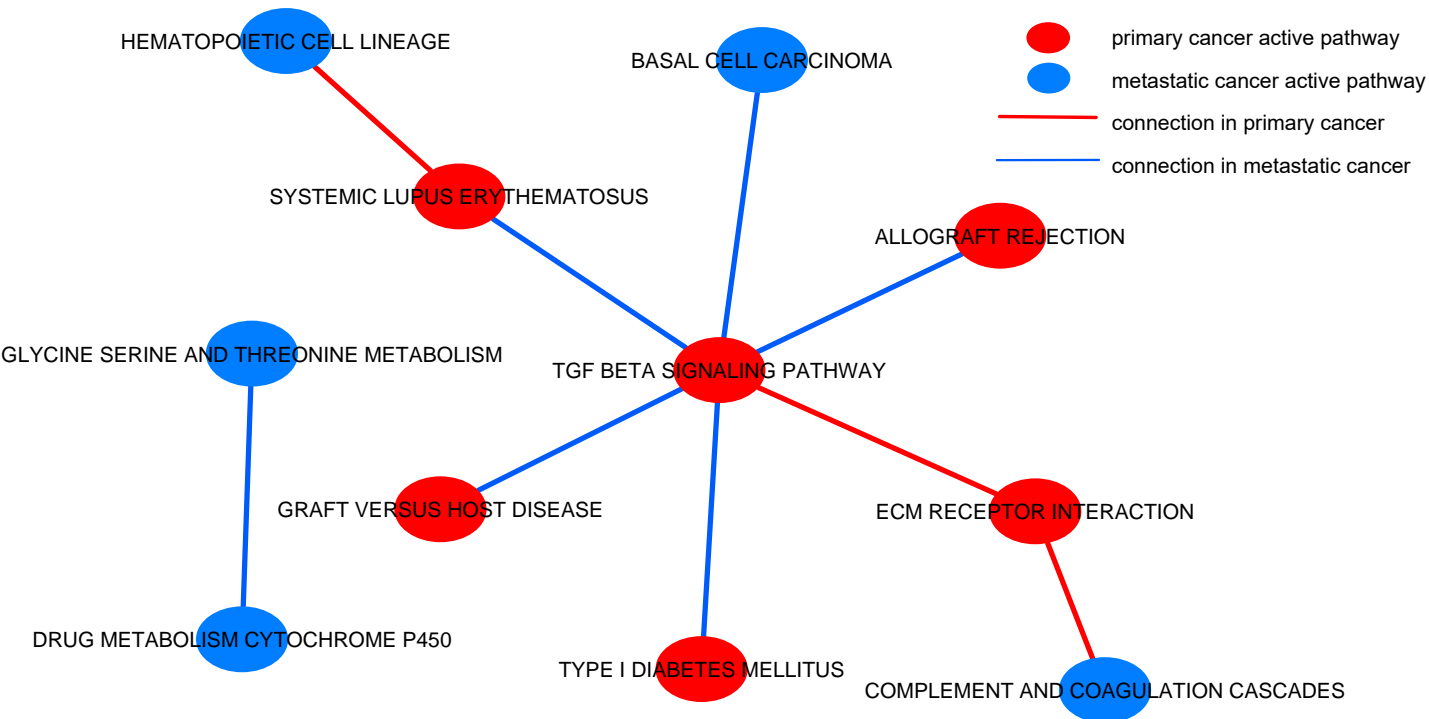

Supplement: Supplementary file 1 [file genes-10-00753-s001.zip › figures and tables final/supplemental figure s7 cancer specific 9 special interaction.pdf]

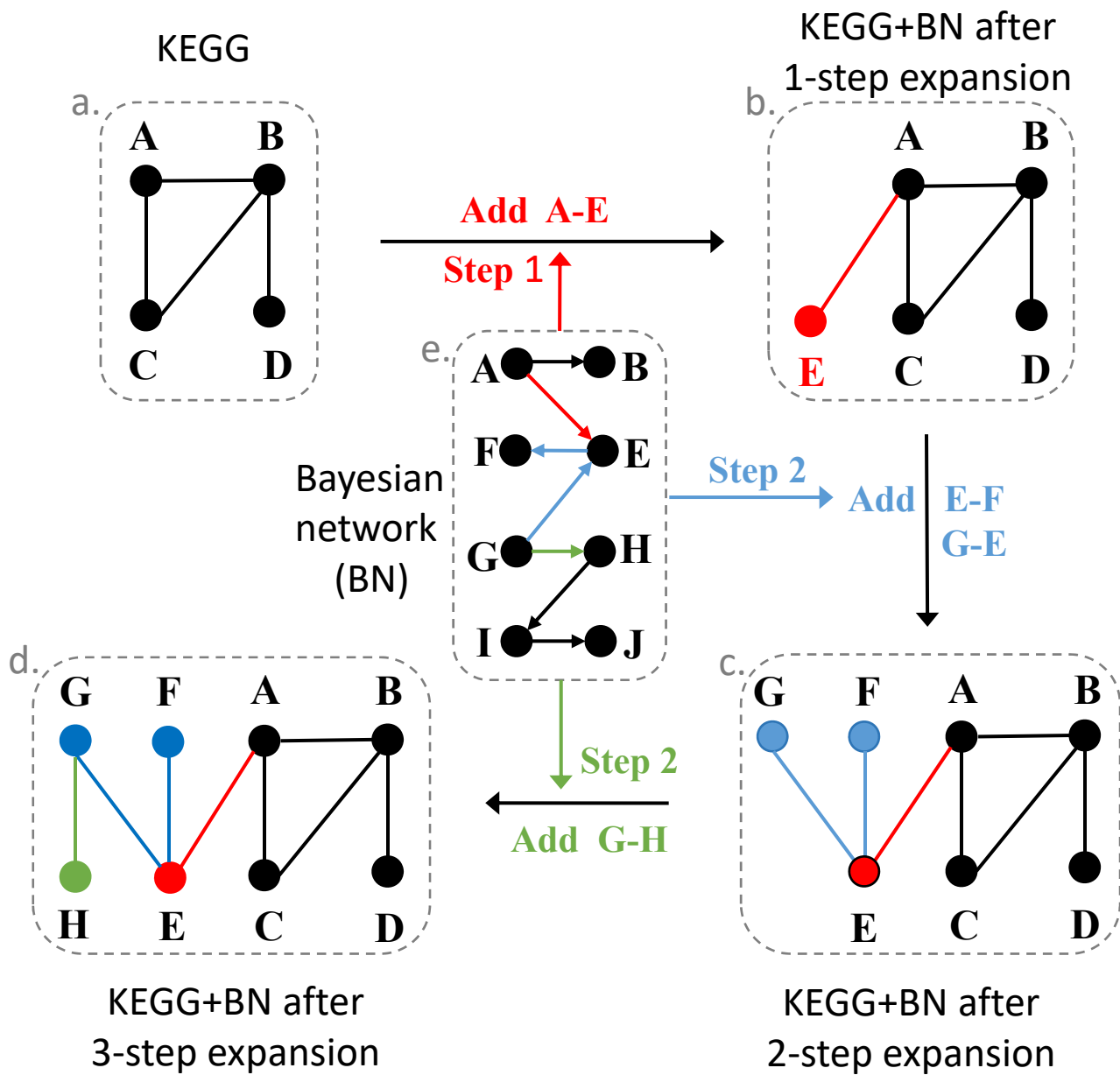

Supplement: Supplementary file 1 [file genes-10-00753-s001.zip › figures and tables final/figure 3 pathway extention.pdf]

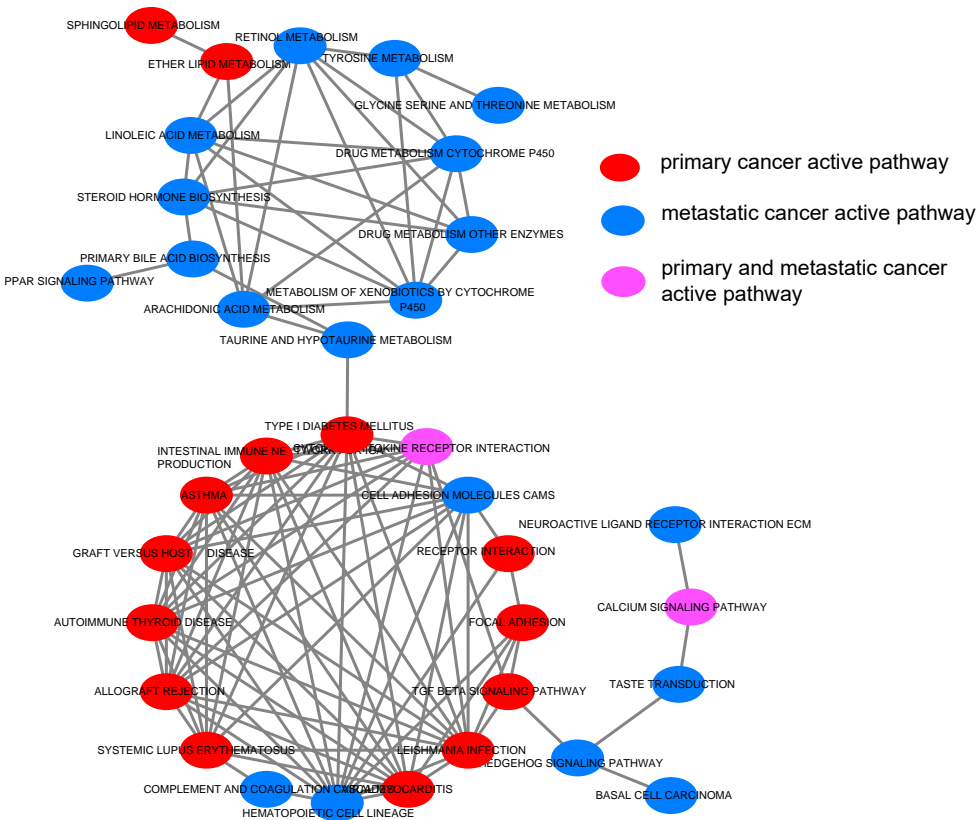

Supplement: Supplementary file 1 [file genes-10-00753-s001.zip › figures and tables final/figure 4 pathway raw 109 new.pdf]

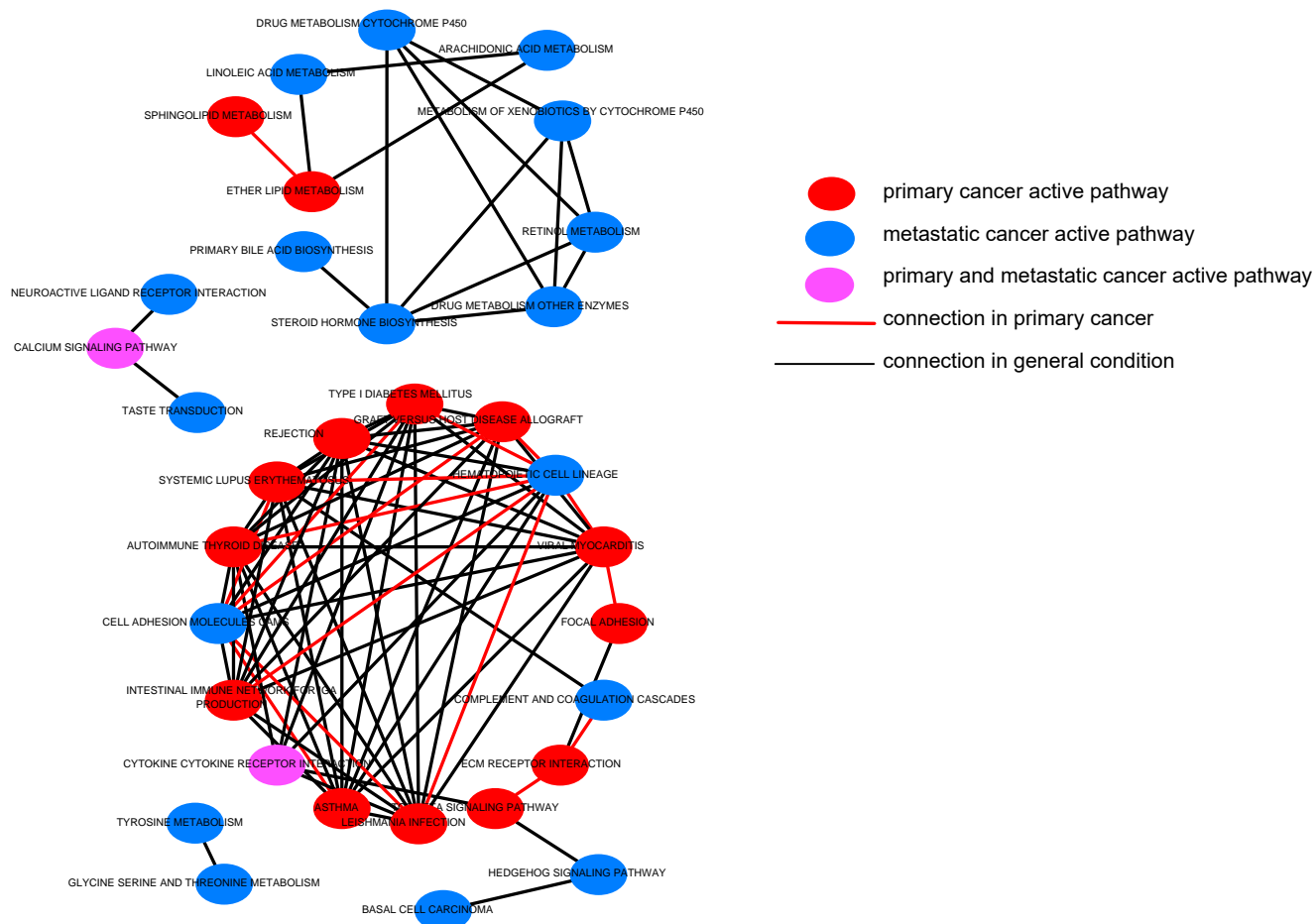

Supplement: Supplementary file 1 [file genes-10-00753-s001.zip › figures and tables final/supplemental figure s2 pathway_common_and_seprate_86 primary.pdf]

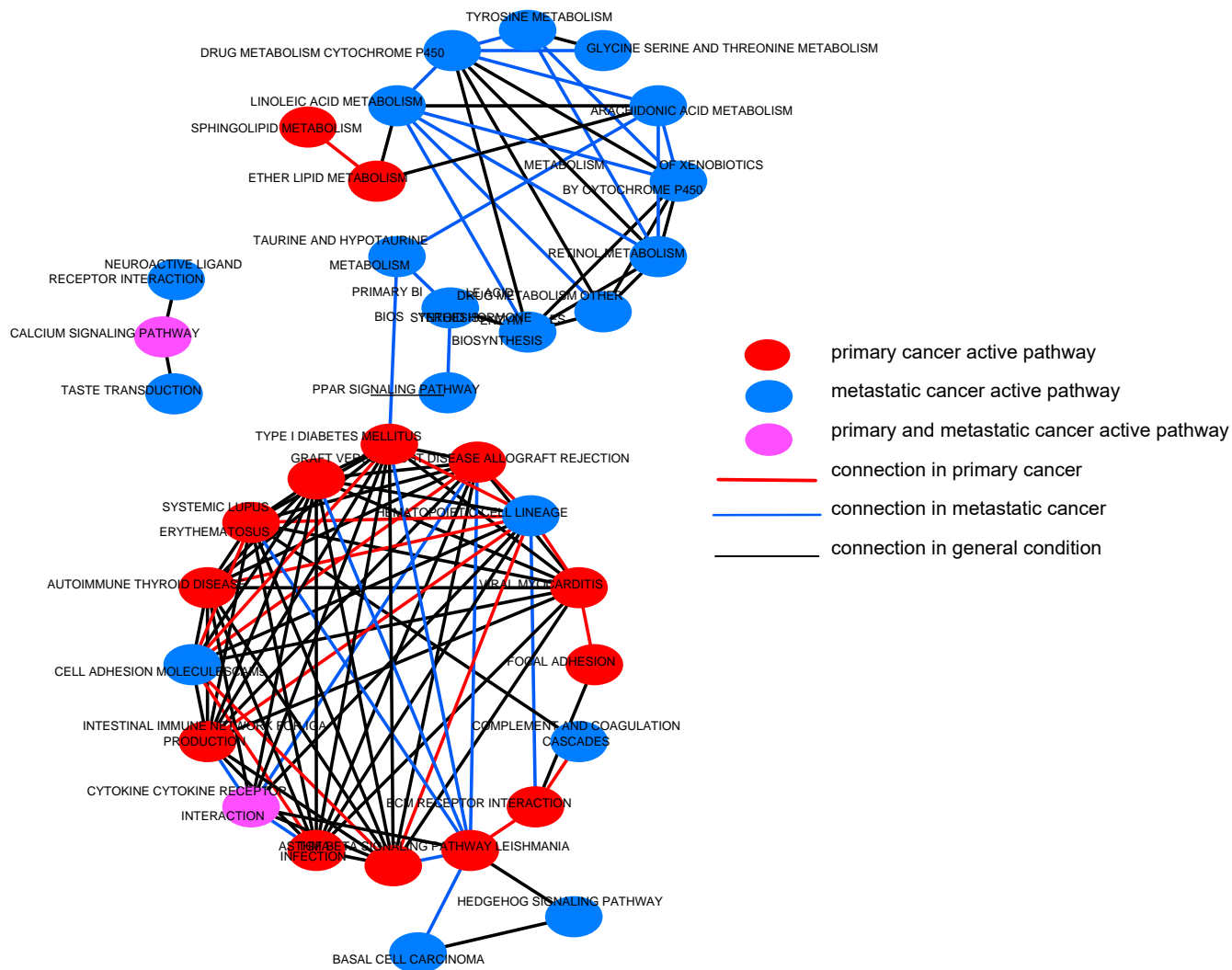

Supplement: Supplementary file 1 [file genes-10-00753-s001.zip › figures and tables final/supplemental figure s4 pathway_common_and_seprate_112_primary and metastasis.pdf]

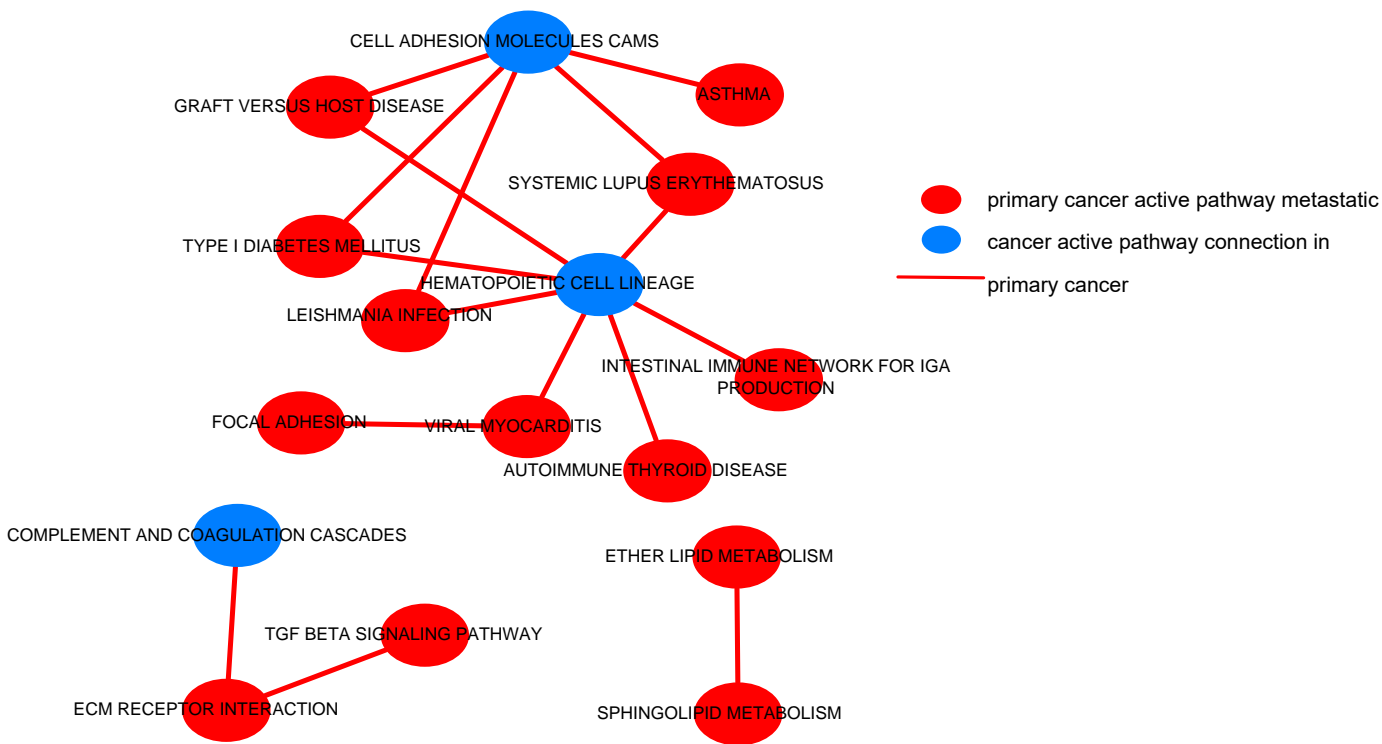

Supplement: Supplementary file 1 [file genes-10-00753-s001.zip › figures and tables final/supplemental figure 5 primay specific 16.pdf]

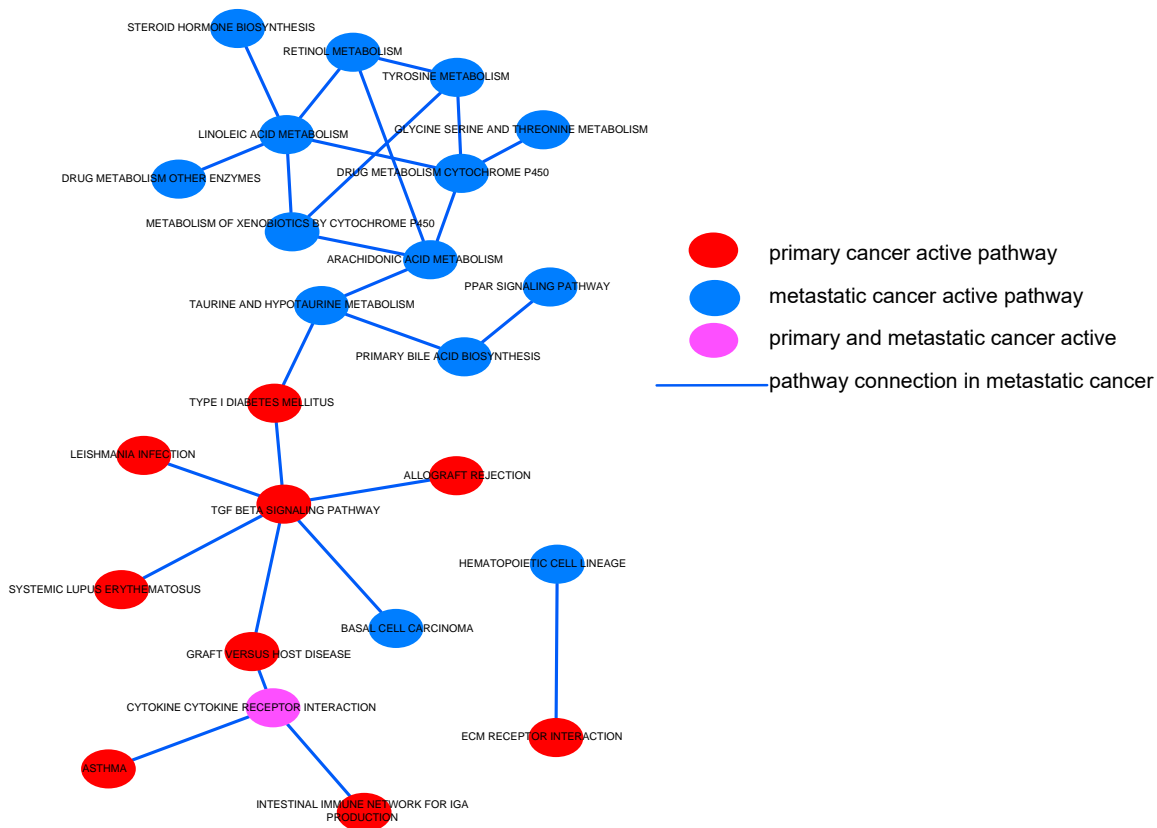

Supplement: Supplementary file 1 [file genes-10-00753-s001.zip › figures and tables final/supplemental figure s6 metastatic specific 26.pdf]

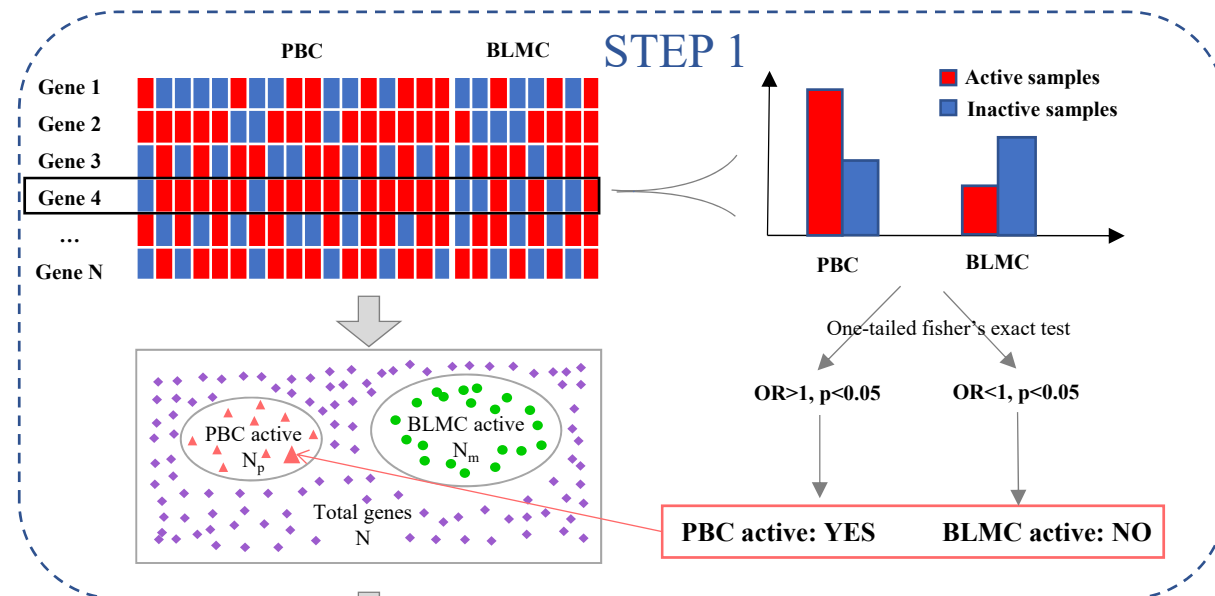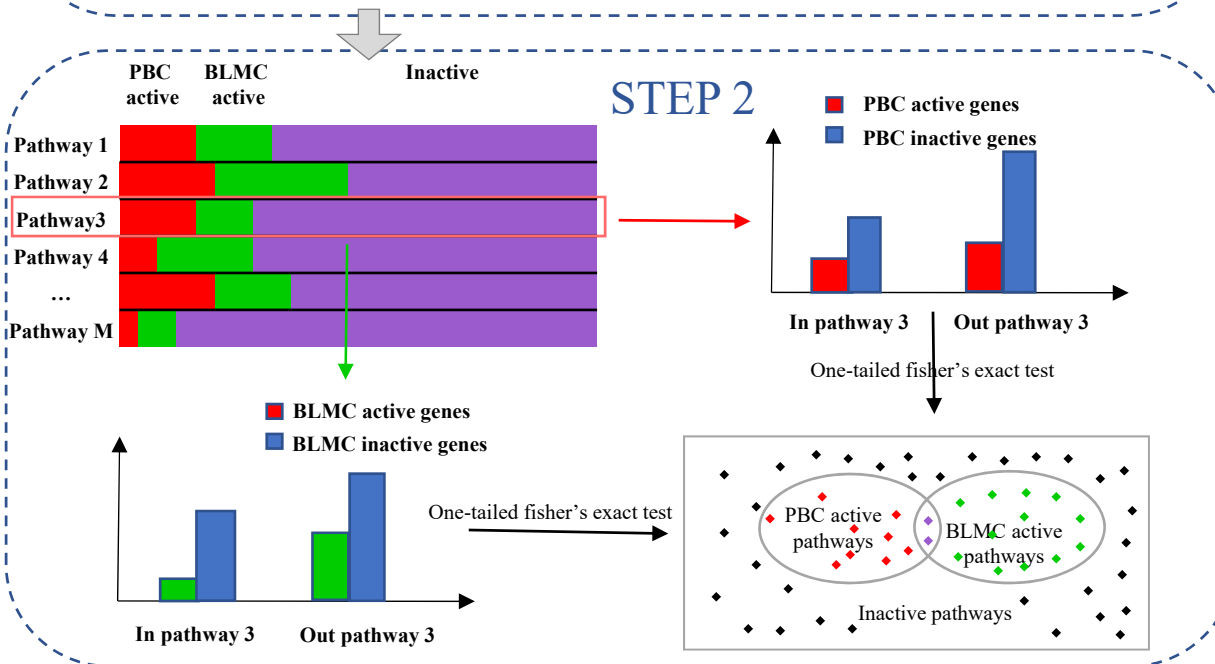

Supplement: Supplementary file 1 [file genes-10-00753-s001.zip › figures and tables final/figure 1 flowchart_new.pdf]
